# Hospital Nurse Understaffing and Patient Mortality, Readmission, and Length of Stay

**DOI:** 10.1001/jamanetworkopen.2025.58235

**Published:** 2026-02-25

**Authors:** Noriko Morioka, Mutsuko Moriwaki, Atsushi Miyawaki, Christina Saville, Kiyohide Fushimi, Peter Griffiths

**Affiliations:** 1 Department of Health Policy and Informatics, Graduate School of Medical and Dental Sciences, Institute of Science Tokyo, Tokyo, Japan; 2 Department of Epidemiology and Biostatistics, National Institute of Public Health, Saitama, Japan; 3 Quality Management Center, Institute of Science Tokyo, Tokyo, Japan; 4 Public Health Research Group, Institute of Medicine, University of Tsukuba, Tsukuba, Japan; 5 Health Workforce and Systems, School of Health Sciences, University of Southampton, Southampton, United Kingdom

## Abstract

**Importance:**

Although higher nurse staffing has been linked to better patient outcomes, most studies rely on aggregate measures, leaving the effects of deviations from ward- and shift-level norms (daily and per shift) unclear. Clarifying these effects can inform ward-level staffing decisions.

**Objective:**

To investigate the association between nurse understaffing, relative to typical ward staffing levels, during the 24-hour period, day shift, and evening and night shift and patient risk for in-hospital mortality, readmission, and longer length of stay (LOS).

**Design, Setting, and Participants:**

This retrospective cohort study used claims data and shift-ward rosters from 82 wards in 9 hospitals in Japan. Included patients were aged 20 years or older and hospitalized between April 1, 2019, and March 31, 2020. Analyses were performed from March 20 to November 12, 2025.

**Exposure:**

Nurse understaffing was defined as average nurse hours per patient-day below each ward’s annual median, assessed for the 24-hour period, day shift, and evening and night shift.

**Main Outcomes and Measures:**

The primary outcomes assessed were in-hospital mortality, 7-day and 30-day readmissions, and LOS. Propensity score matching (PSM) and multilevel models adjusted for confounding.

**Results:**

This study included 77 289 hospital admissions. Patients had a mean (SD) age of 69.3 (15.1) years, 57.2% were male, and 53.2% were admitted for surgery. Of these admissions, 28 846 matched pairs were included in the in-hospital mortality analysis and 27 907 matched pairs were included in the readmission analysis. After PSM, patients exposed to nurse understaffing during the 24-hour period and day shift had higher in-hospital mortality rates (3.1% vs 2.8% and 3.2% vs 2.8%; both *P* = .02); those exposed to understaffing during the 24-hour period also had higher 30-day readmission rates (11.2% vs 10.5%; *P* = .01), whereas those exposed to understaffing during the day shift had higher 7-day readmission rates (2.3% vs 2.1%; *P* = .04). Understaffing during the evening and night shift was not associated with these outcomes. LOS after PSM was longer when understaffing occurred during the 24-hour period (mean [SD], 14.6 [16.3] vs 13.8 [16.5] days), day shift (14.7 [16.4] vs 13.7 [16.2] days), or evening and night shift (14.1 [16.2] vs 13.6 [16.4] days) (all *P* < .001).

**Conclusions and Relevance:**

In this cohort study, nurse understaffing during the 24-hour period (specifically during the day shift) was associated with increased risk of in-hospital mortality, hospital readmission, and longer LOS. These findings suggest that close attention to daily staffing levels and prompt action to address understaffing may help improve patient outcomes. Nurse managers should routinely monitor deviations from this benchmark to address immediate needs and enable rapid adjustments through feasible strategies.

## Key Points

**Question:**

Is nurse understaffing during the day shift or evening and night shift, relative to typical ward staffing levels, associated with patient outcomes in acute care hospitals?

**Findings:**

In this cohort study of 77 289 hospital admissions across 82 wards in Japan, patients exposed to nurse understaffing during the day shift or during the 24-hour period had higher in-hospital mortality and increased readmission rates, whereas evening and night shift understaffing was not associated with these outcomes. Understaffing during the 24-hour period, as well as during the day shift and evening and night shift, was associated with longer hospital stays.

**Meaning:**

These findings suggest that maintaining nurse staffing at or above the ward’s typical level for each shift, particularly during day shifts, is associated with improved patient outcomes.

## Teaser

This cohort study explores the association between nurse understaffing during the day shift and evening and night shift, relative to typical ward staffing levels, and patient outcomes in acute care hospitals.

## Introduction

Nurses constitute the largest segment of the health and care workforce, and their effective deployment is critical to the quality and cost efficiency of health care systems.^1^ Over the past 3 decades, robust evidence has demonstrated that higher nurse staffing levels are associated with improved patient outcomes, including reduced mortality and adverse events.^2,3,4^ However, a persistent challenge remains^4,5,6^: The association between adequate nurse staffing and improved patient outcomes is well established, but frontline decisions about appropriate staffing levels are typically made by nurse managers based on their expert judgment. Although various nurse staffing measurement tools exist^7,8^ (eg, RAFAELA [Resource Allocation for Appropriate Fagerström-Andersen Evaluation of Nursing Intensity] in Nordic countries^9^ or the Safer Nursing Care Tool in the UK^10^), these tools are often used in conjunction with professional judgment, and evidence-based guidance to support such decisions is still needed.^7,11^

Several previous studies have used fixed patient-to-nurse ratios (eg, 5:1 vs 6:1) or increases in nursing hours per patient-day (NHPPD) for absolute comparisons.^4^ However, such approaches may not adequately account for the variability in care demands across hospital units, where optimal staffing levels differ based on clinical context.^12^ To address these limitations, some studies have adopted models that evaluate deviations from typical ward-level staffing.^13,14,15,16,17^ Using the ward mean or median as a reference, adequate staffing has been associated with lower mortality and readmission rates as well as shorter length of stay (LOS), whereas understaffing has been linked to an increased risk of adverse outcomes.

Despite the aforementioned advances, most existing studies rely on aggregated 24-hour staffing^13,14,15,17^ or shift-level staffing data without distinguishing shifts by time of day,^16,18,19^ which may obscure important variations in care delivery across shifts. In clinical practice, nursing care is organized into distinct day and night shifts,^20^ and shift-specific staffing levels may independently influence patient outcomes. Few studies have shown that higher nurse staffing, when distinguishing between day and night shifts, is associated with lower mortality^18^ and a reduced risk of hospital-associated disability among older adults.^21^ Disaggregated, shift-level analyses provide more granular and actionable insights for nurse managers and hospital administrators aiming to make timely, data-informed staffing decisions.

A key limitation of prior observational studies is the difficulty in establishing causal relationships between nurse staffing levels and patient outcomes.^5,6^ Although randomized clinical trials are ideal for causal inference, they are often infeasible or ethically inappropriate in this context. Consequently, there is increasing emphasis on the use of more robust analytic approaches, such as propensity score methods, which improve covariate balance and restrict analyses to the region of common support, thereby enhancing the validity of causal inference in nurse staffing research. This study aimed to examine the association between nurse understaffing—defined relative to typical ward-level staffing for the whole day (24-hour period) as well as for individual day and night shifts—and in-hospital mortality, hospital readmission, and LOS among adults in acute care settings, using propensity score matching (PSM).

## Methods

This retrospective cohort study was approved by the Institute of Science Tokyo. The study adhered to the Declaration of Helsinki,^22^ and the requirement for informed consent was waived due to the use of anonymized data. We followed the Strengthening the Reporting of Observational Studies in Epidemiology (STROBE) and the Reporting of Studies Conducted Using Observational Routinely-Collected Health Data (RECORD) reporting guidelines.

### Setting and Data Sources

We used linked data comprising patient discharge records and daily ward-level nurse staffing rosters from 82 general medical or surgical wards across 9 hospitals affiliated with the National Hospital Organization (NHO) in Japan. Patient data were derived from the Diagnosis Procedure Combination (DPC) database, a national case-mix classification and bundled reimbursement system. The DPC database includes patient demographic information (age, sex, and body mass index), major diagnostic categories (MDCs), dates and status (eg, consciousness level, ambulance use, and prehospitalization residence), and discharge dates and discharge status (eg, in-hospital death or postdischarge residence). Additional details have been described previously.^23,24^

Nurse staffing data were extracted from a standardized administrative dataset (form no. 9) that is mandated under the National Health Insurance fee schedule. This dataset reports total daily nursing hours per shift (day or evening and night). In Japan, staffing levels are regulated and linked to reimbursement tiers.^25^ Hospitals are mandated to submit nurse roster data for each ward to the Branch of the Ministry of Labour, Health, and Welfare, including information for the day shift (8 hours) and the combined evening and night shifts (16 hours, covering 10:00 pm to 2:00 am). All hospitals in this study applied the highest staffing level for general wards (7:1 patient-to-nurse ratio, equivalent to approximately 3.4 NHPPD).^25^ We linked the individual DPC data with ward-level nurse staffing data using the date and ward identification number (ID) as key variables.

### Study Population

This study included patients aged 20 years or older who were admitted to and discharged from one of the participating hospitals between April 1, 2019, and March 31, 2020. Patients were excluded if they died within 24 hours of admission, had planned repeated readmissions, were admitted for educational purposes, or could not be linked to the ward-level staffing data due to multiple ward transfers (eFigure 1 in Supplement 1).

### Variables

#### Exposure

The exposure variable was nurse understaffing. For each patient, nurse understaffing during hospitalization (excluding days spent in the intensive care unit [ICU]) was defined as an average nursing staffing level, measured in NHPPD, that fell below the ward-specific annual median NHPPD. Nurse understaffing was defined in 2 ways: (1) based on the ward-specific annual median NHPPD for the 24-hour period and (2) based on the annual median NHPPD calculated separately for the day shift (9:00 am to 5:00 pm; 8 hours) and evening and night shift (5:00 pm to 9:00 am; 16 hours). Although prior studies relying solely on 24-hour averages may obscure shift-level variation, examining aggregate and shift-specific staffing allows for assessment of whether daily patient outcomes are driven by specific shifts. Associations with patient outcomes were examined using 3 separate models reflecting these definitions. This relative approach, based on deviations from ward-level annual medians, has been used in a prior study^16,17^ and reflects the variability in optimal staffing needs depending on patient acuity and care complexity.

In Japan, the qualifications for nurses and associate nurses are defined under the Act on Public Health Nurses, Midwives, and Nurses.^26^ Registered nurses are certified through a national licensing examination, whereas associate nurses obtain their licenses from prefectural governors. However, both types of licenses are valid for practice nationwide.^26^ According to the Ministry of Health, Labour and Welfare annual hospital report,^27^ as of July 2019, all staff working in the study wards were nurses; no associate nurses were employed. Each ward had, on average, 1.5 full-time equivalent assistant nurses, although daily data for these staff were unavailable.

#### Outcomes

We evaluated 3 patient outcomes: in-hospital mortality, unplanned readmission within 7 or 30 days of discharge to the same hospital, and LOS. Planned readmissions for chemotherapy or radiation therapy, educational admissions, or diagnostic procedures were excluded. These outcomes have been validated in prior umbrella reviews as sensitive to nursing care.^5^

#### Adjustment Variables

Adjustment variables were selected based on prior studies^13,14,15,16,17,18^ and expert consensus. Patient-level variables included age, sex, body mass index, smoking exposure (using the Brinkman Index score,^28^ calculated as the number of cigarettes per day multiplied by years of smoking), Charlson Comorbidity Index score,^29^ unconsciousness at admission, ambulance use at admission, weekend admission, MDC, ICU stay during hospitalization (accounting for patient illness severity and underlying risk or high-risk procedures), surgical compared with medical admission, prehospitalization and postdischarge residence, and illness severity (based on the patient’s score on the Severity of a Patient’s Condition and Extent of a Patient’s Need for Medical/Nursing Care tool^30^). Ward-level variables included total inpatient volume, mean patient age, and proportion of patients classified as high acuity based on the severity score used for high-care unit designation. We also adjusted for indicators of hospitals (hospital fixed effects) to effectively compare patients within the same hospitals. Details on the covariates are presented in eTable 1 in Supplement 1.

### Statistical Analysis

First, patient characteristics were compared between the understaffed and adequately staffed groups as well as between the analytic sample and those excluded because of death within 24 hours of admission (eTable 2 in Supplement 1) or missing data (eTable 3 in Supplement 1). Comparisons were conducted using χ^2^ tests for categorical variables and *t* tests for continuous variables.

Second, annual median NHPPD values were summarized across all 82 wards, and the actual NHPPD during hospitalization was calculated for the 24-hour period, day shift, and evening and night shift. Third, a PSM analysis, a quasi-experimental design that balances baseline characteristics between groups, was conducted. Model 1 examined 24-hour staffing, model 2 assessed the day shift, and model 3 evaluated the evening and night shift. For the readmission models, patients who died during the index hospitalization were excluded.

Third, in the propensity score estimation, the aforementioned adjustment variables were included, along with hospital IDs as fixed effects (sets of dummy variables), to account for clustering at the hospital level. Matching was performed using 1:1 nearest-neighbor matching with replacement and a caliper width of 0.2 of the SD of the logit of the propensity score. Model discrimination was assessed using the C statistic (ie, the area under the receiver operating characteristic curve) from logistic regression models. C statistics ranged from 0.69 to 0.85; values greater than 0.7 were considered acceptable. Covariate balance between the treatment (understaffed) and control (adequately staffed) groups after PSM was assessed using standardized mean differences (SMDs) for each covariate. An SMD of 0.1 or less was considered indicative of negligible imbalance, suggesting successful covariate adjustment and effective baseline comparability.

Fourth, multilevel random-effects regression analyses using a 3-level structure (individual nested within wards, nested within hospitals) were conducted to examine the associations between nurse understaffing and patient outcomes. Logistic regression was used for in-hospital mortality and readmission, and linear regression was used for LOS, adjusting for confounders. Hospital IDs were included as fixed effects, with random intercepts specified at both the ward and hospital levels.

Fifth, as a sensitivity analysis, we performed PSM and multilevel analyses that included planned repeated readmission cases for all 3 models and outcomes and excluded in-hospital deaths from the analysis of LOS. We also repeated the PSM and multilevel logistic regression using 6 alternative NHPPD cutoff points for defining understaffing: 15% (approximately 5.3 NHPPD), 10%, 5%, −5%, −10%, and −15% (approximately 3.5 NHPPD) relative to the annual ward-level median. These values align with the operational range under Japan’s 7:1 patient-to-nurse ratio (equivalent to 3.4 NHPPD).

All tests were 2 sided, and *P* < .05 was considered statistically significant. Analyses were conducted from March 20 to November 12, 2025, using Stata, version 18 MP (StataCorp LLC).

## Results

### Patient Characteristics

A total of 77 289 eligible hospital admissions were identified (eFigure 1 in Supplement 1). Patients had a mean (SD) age of 69.3 (15.1) years; 33 075 (42.8%) were female and 44 214 (57.2%) were male. More than half of patients (41 137 [53.2%]) were admitted for surgery. The most common MDC was diseases and disorders of the digestive system, hepatobiliary system, and pancreas, comprising 16 688 admissions (21.6%). Baseline characteristics before and after PSM are presented in Table 1 and eTables 4 to 10 in Supplement 1.

**Table 1.** Patient Characteristics Before and After PSM for 24-Hour Nurse Understaffing Using the Annual Median as the Cutoff Point^a^

Characteristic	Before PSM			After PSM for death^b^		
	Adequately staffed group (n = 43 770)	Understaffed group (n = 33 516)	SMD	Adequately staffed group (n = 28 846)	Under staffed group (n = 28 846)	SMD (95% CI)
Sex						
Female	18 599 (42.5)	14 476 (43.2)	−0.01	12 396 (43.0)	12 161 (42.2)	−0.01
Male	25 171 (57.5)	19 040 (56.8)	0.01	16 450 (57.0)	16 685 (57.8)	0.01
Age, mean (SD), y	69.1 (15.0)	69.4 (15.3)	−0.02	69.4 (15.2)	69.3 (15.0)	−0.01
BMI, mean (SD)	23.1 (4.1)	23.1 (4.2)	0.02	23.1 (4.2)	23.1 (4.2)	0.005
CCI score						
0	21 795 (49.8)	15 992 (47.7)	0.04	13 848 (48.0)	13 917 (48.2)	0
1	3378 (7.7)	2654 (7.9)	−0.01	2294 (8.0)	2259 (7.8)	−0.004
2	12 593 (28.8)	10 017 (29.9)	−0.02	8504 (29.5)	8542 (29.6)	0.004
≥3	6004 (13.7)	4853 (14.5)	−0.02	4200 (14.6)	4128 (14.3)	−0.002
Smoking exposure (Brinkman Index score^c^)						
0	22 402 (51.2)	17 413 (52.0)	−0.02	14 942 (51.8)	14 778 (51.2)	−0.01
1-399	4716 (10.8)	3515 (10.5)	0.01	3026 (10.5)	3078 (10.7)	0.004
400-599	2694 (6.2)	2123 (6.3)	−0.01	1792 (6.2)	1811 (6.3)	0.005
≥600	13 958 (31.9)	10 465 (31.2)	0.01	9086 (31.5)	9179 (31.8)	0.002
Prehospitalization residence						
Home	41 763 (95.4)	31 714 (94.6)	0.04	27 348 (94.8)	27 398 (95.0)	0.01
Hospital or clinic	1112 (2.5)	977 (2.9)	−0.02	810 (2.8)	801 (2.8)	−0.01
Long-term care	895 (2.0)	825 (2.5)	−0.03	688 (2.4)	647 (2.2)	−0.01
Unconsciousness at admission	3923 (9.0)	3798 (11.3)	−0.08	2993 (10.4)	2831 (9.8)	−0.02
Weekend admission	4991 (11.4)	4329 (12.9)	−0.05	3580 (12.4)	3390 (11.8)	−0.01
Surgery	23 054 (52.7)	18 083 (54.0)	−0.03	15 448 (53.6)	15 421 (53.5)	−0.003
Ambulance use at admission	6917 (15.8)	6653 (19.9)	−0.11	5344 (18.5)	4877 (16.9)	−0.02
ICU experience	1122 (2.6)	974 (2.9)	−0.02	804 (2.8)	838 (2.9)	−0.002

Abbreviations: BMI, body mass index (calculated as weight in kilograms divided by height in meters squared); CCI, Charleson Comorbidity Index; ICU, intensive care unit; PSM, propensity score matching; SMD, standardized mean difference.

^a^ Unless otherwise indicated, values are presented as No. (%) of patients. Adequately staffed indicates staffing levels at or greater than the annual median, whereas understaffed indicates staffing levels less than the annual median.

^b^ Adjusted for age, age squared, CCI score, smoking exposure (Brinkman Index score), BMI, prehospitalization residence, diagnosis, ICU stay, surgery, unconsciousness at admission, ambulance use, weekend admission, illness severity at admission (score on the Severity of a Patient’s Condition and Extent of a Patient’s Need for Medical/Nursing Care tool), percentage of inpatients with severe illness on a ward, median inpatient age on a ward, and number of inpatients on a ward, and hospital identification number (dummy variable).

^c^ Calculated as cigarettes per day multiplied by years of smoking.

Before PSM, there were 43 770 patients in the adequately staffed group and 33 516 in the understaffed group. Most SMDs were minimal, and moderate imbalance was noted for ambulance use at admission (−0.11) and unconsciousness at admission (−0.08). After 1:1 PSM was performed separately for in-hospital mortality and readmission, 28 846 matched pairs were included in the mortality analysis and 27 907 matched pairs were included in the readmission analysis. Covariate balance was achieved (with all SMDs <±0.02). After PSM, the mean (SD) patient age (adequately staffed, 69.4 [15.2] vs understaffed, 69.3 [15.0] years), proportion of female patients (adequately staffed, 43.0% vs understaffed, 42.2%), comorbidity burden, and other clinical characteristics were comparable between groups. The largest residual imbalance was for ambulance use at admission (−0.02), which was negligible.

### Nurse Staffing Levels

Across the 82 wards, the overall mean (SD) NHPPD was 4.40 (0.88), 2.60 (0.51), and 1.79 (0.42) for the 24-hour period, day shift, and evening and night shift, respectively (Table 2 and eFigure 2 in Supplement 1). Before and after PSM, the mean (SD) 24-hour NHPPD during hospitalization was 4.73 (0.84) and 4.53 (0.72) in the adequately staffed group and 4.00 (0.66) and 4.06 (0.68) in the understaffed group, using the annual median as the cutoff. Distributions under alternative cutoff thresholds are presented in eTables 4 to 10 in Supplement 1.

**Table 2.** Annual Ward-Level Nurse Staffing and Actual Nurse Staffing

Staffing by model	Nursing hours per patient-day, mean (SD)	Percentile		
		50th	25th	75th
Annual ward-level nurse staffing (n = 82 wards)				
24-h Period	4.40 (0.88)	4.11	3.82	4.76
Day shift	2.60 (0.51)	2.46	2.24	2.88
Evening and night shift	1.79 (0.42)	1.69	1.54	1.92
Actual nurse staffing during hospitalization				
24-h Period (n = 77 286 patients)	4.41 (0.85)	4.25	3.82	4.83
Adequately staffed group (n = 43 770)	4.73 (0.84)	4.54	4.13	5.16
Understaffed group (n = 33 516)^a^	4.00 (0.66)	3.87	3.56	4.28
Day shift	2.65 (0.57)	2.55	2.23	2.96
Adequately staffed group	2.88 (0.55)	2.77	2.47	3.18
Understaffed group	2.34 (0.44)	2.26	2.03	2.57
Evening and night shift	1.77 (0.35)	1.71	1.55	1.93
Adequately staffed group	1.85 (0.37)	1.79	1.61	2.03
Understaffed group	1.67 (0.30)	1.63	1.49	1.80

^a^ Understaffing was defined as nursing hours per patient-day below the annual ward median during the 24-hour period, day shift, or evening and night shift.

### Patient Outcomes

In the full sample, unadjusted in-hospital mortality was higher in the understaffed group compared with the adequately staffed group during the 24-hour period (3.3% vs 2.5%; *P* < .001), day shift (3.3% vs 2.5%; *P* < .001), and evening and night shift (2.9% vs 2.8%; *P* = .22) before PSM (Table 3). Similar patterns were observed after PSM, with higher mortality in the understaffed group during the 24-hour period (3.1% vs 2.8%; *P* = .02) and during the day shift (3.2% vs 2.8%; *P* = .02). No significant differences were observed for the evening and night shift (3.1% vs 3.0%; *P* = .50).

**Table 3.** Comparison of Patient Outcomes Between Adequately Staffed and Understaffed Groups Before and After PSM^a^

Outcome by model	Before PSM			After PSM^b^		
	Adequately staffed group	Understaffed group	*P* value^c^	Adequately staffed group	Understaffed group	*P* value^c^
In-hospital death						
24-h Period	1094/43 770 (2.5)	1110/33 516 (3.3)	<.001	807/28 846 (2.8)	905/28 846 (3.1)	.02
Day shift	1104/44 409 (2.5)	1100/32 877 (3.3)	<.001	884/31 030 (2.8)	983/31 030 (3.2)	.02
Evening and night shift	1141/41 014 (2.8)	1063/36 272 (2.9)	.22	722/24 462 (3.0)	750/24 462 (3.1)	.50
7-d Readmission						
24-h Period	890/42 676 (2.1)	735/32 406 (2.3)	.09	616/27 907 (2.2)	636/27 907 (2.3)	.57
Day shift	883/43 305 (2.0)	742/31 777 (2.3)	.01	628/29 999 (2.1)	701/29 999 (2.3)	.04
Evening and night shift	845/39 873 (2.1)	780/35 209 (2.2)	.37	538/23 737 (2.3)	529/23 737 (2.2)	.78
30-d Readmission						
24-h Period	4643/42 676 (10.9)	3539/32 406 (10.9)	.86	2942/27 907 (10.5)	3126/27 907 (11.2)	.01
Day shift	4724/43 305 (10.9)	3458/31 777 (10.9)	.91	3169/29 999 (10.6)	3306/29 999 (11.0)	.07
Evening and night shift	4375/39 873 (11.0)	3807/35 209 (10.8)	.48	2601/23 737 (11.0)	2637/23 737 (11.1)	.60
Length of stay, mean (SD), d						
24-h Period	12.6 (15.2) (n = 43 770)	15.1 (17.1) (n = 33 516)	<.001	13.8 (16.5) (n = 28 846)	14.6 (16.3) (n = 28 846)	<.001
Day shift	12.6 (15.1) (n = 31 777)	15.1 (17.1) (n = 31 777)	<.001	13.7 (16.2) (n = 31 030)	14.7 (16.4) (n = 31 030)	<.001
Evening and night shift	13.2 (16.0) (n = 35 209)	14.1 (16.2) (n = 35 209)	<.001	13.6 (16.4) (n = 36 272)	14.1 (16.2) (n = 36 272)	<.001

Abbreviation: PSM, propensity score matching.

^a^ Unless indicated otherwise, values are reported as No. of patients/Total No. of patients (%). Adequately staffed indicates staffing levels at or greater than the annual median, whereas understaffed indicates staffing levels less than the annual median.

^b^ Adjusted for age, age squared, Charlson Comorbidity Index score, smoking exposure (Brinkman Index score), body mass index, prehospitalization residence, diagnosis, intensive care unit stay, surgery, unconsciousness at admission, ambulance use, weekend admission, illness severity at admission (score on the Severity of a Patient’s Condition and Extent of a Patient’s Need for Medical/Nursing Care tool), percentage of inpatients with severe illness on a ward, median inpatient age on a ward, number of inpatients on a ward, and hospital identification number (dummy variable).

^c^ χ^2^ test or *t* test.

After PSM, 7-day readmission rates did not differ significantly between groups for the 24-hour period or the evening and night shift. However, patients who experienced nurse understaffing during the day shift had higher 7-day readmission rates compared with those in the adequately staffed group (2.3% vs 2.1%; *P* = .04). For 30-day readmission, rates were significantly higher in the understaffed group during the 24-hour period (11.2% vs 10.5%; *P* = .01), with no significant differences during the day shift (11.0% vs 10.6%; *P* = .07) or evening and night shift (11.1% vs 11.0%; *P* = .60). LOS was significantly longer in the understaffed group across the 24-hour period, day shift, and evening and night shift before PSM (mean [SD], 15.1 [17.1] vs 12.6 [15.2] days, 15.1 [17.1] vs 12.6 [15.1] days, and 14.1 [16.2] vs 13.2 [16.0] days, respectively) and after PSM (mean [SD], 14.6 [16.3] vs 13.8 [16.5] days, 14.7 [16.4] vs 13.7 [16.2] days, and 14.1 [16.2] vs 13.6 [16.4] days, respectively) (all *P* < .001) (Table 3).

### Multilevel Analysis of Nurse Understaffing and Patient Outcomes

Multilevel logistic regression models, incorporating random intercepts for wards and hospitals, yielded results consistent with the PSM analyses (Table 4). After adjustment, nurse understaffing during the 24-hour period was associated with increased odds of in-hospital mortality (adjusted odds ratio [AOR], 1.22 [95% CI, 1.11-1.34]), 30-day readmission (AOR, 1.05 [95% CI, 1.00-1.10]), and longer LOS (adjusted coefficient, 1.22 [95% CI, 1.00-1.43]).

**Table 4.** Multilevel Analysis of Nurse Understaffing and Patient Outcomes^a^

Outcome by model	Odds ratio or coefficient (95% CI)^b^	*P* value
In-hospital death		
24-h Period	1.22 (1.11-1.34)	<.001
Day shift	1.20 (1.10-1.32)	<.001
Evening and night shift	1.04 (0.94-1.15)	.43
7-d Readmission		
24-h Period	1.07 (0.96-1.18)	.22
Day shift	1.12 (1.01-1.24)	.03
Evening and night shift	1.04 (0.94-1.16)	.47
30-d Readmission		
24-h Period	1.05 (1.00-1.10)	.05
Day shift	1.04 (0.99-1.10)	.08
Evening and night shift	1.00 (0.94-1.05)	.91
Length of stay		
24-h Period	1.22 (1.00-1.43)	<.001
Day shift	1.22 (1.01-1.44)	<.001
Evening and night shift	−0.16 (−0.40 to 0.09)	.21

^a^ Understaffing was defined as staffing levels below the annual ward median during the 24-hour period, day shift, or evening and night shift.

^b^ Odds ratios are reported for in-hospital death, 7-day readmission, and 30-day readmission, and coefficients are reported for length of stay. Values were adjusted for age, age squared, Charlson Comorbidity Index score, smoking exposure (Brinkman Index score), body mass index, prehospitalization residence, intensive care unit stay, unconsciousness at admission, ambulance use, weekend admission, residence after discharge (only readmission model), main diagnosis combination, illness severity at admission (scores for items A, B, and C on the Severity of a Patient’s Condition and Extent of a Patient’s Need for Medical/Nursing Care tool), percentage of inpatient with severe illness on a ward, median inpatient age on a ward, number of inpatients on a ward, and hospital identification number (dummy variable).

### Sensitivity Analyses

No material differences were observed in analyses including planned repeated readmission cases (eTables 11 and 12 in Supplement 1) or excluding in-hospital deaths from the analysis of LOS (eTables 13 and 14 in Supplement 1). In sensitivity analyses using alternative cutoff points, in-hospital mortality and LOS remained notably higher in the understaffed group across all models. Substantial differences in 7-day and 30-day readmissions were observed only at cutoff points above the median (5% and 10%) and only in models based on 24-hour period or day-shift staffing (eTables 15 and 16 in Supplement 1).

## Discussion

This cohort study, using large-scale, multihospital Japanese administrative and nurse rostering data and employing robust methods such as PSM and multilevel modeling, aimed to examine the association between nurse understaffing (defined as staffing levels below the ward-specific annual median, calculated separately for the day shift and evening and night shift [in addition to the 24-hour period]) and key patient outcomes. Our findings suggest that nurse understaffing relative to typical ward levels was associated with increased in-hospital mortality, higher 30-day readmission rates, and longer LOS, particularly during the day shift and throughout the 24-hour period. These associations remained consistent across PSM and multilevel regression models, supporting the robustness of the findings. To our knowledge, this is the first study to detect these associations using a more robust analytic approach based on large-scale, patient-level administrative data and to demonstrate that results obtained using PSM were qualitatively similar to those obtained using traditional regression models. This alignment enhances confidence in the observed associations and supports the validity of the findings.

The findings of this study are consistent with previous studies from England,^13,14,15^ the US,^16^ Finland,^17^ and Switzerland^18^ that examined daily nurse staffing levels (in NHPPD) and their associations with patient outcomes based on deviations from typical ward-specific levels. For example, Fogg et al^13^ reported that a 0.5-unit increase in NHPPD above the ward mean was associated with a 10% reduction in mortality and a 6% reduction in 30-day readmissions among 9643 inpatients aged 75 years or older in a single English hospital. Meredith et al^15^ used data from 213 910 inpatients across 3 NHS hospital trusts, and they found that exposure to registered nurse understaffing (below the ward mean) was associated with increased risk of mortality, readmission, and longer LOS. The underlying mechanism is that low nurse staffing levels have been associated with missed nursing care,^31,32^ which in turn may lead to adverse patient outcomes such as increased mortality and longer hospital stays, as well as inadequate discharge preparation resulting in higher rates of readmission.

In addition to the use of PSM analysis, a key strength of this study was the stratification of nurse staffing levels by shift (24-hour period, day shift, or evening and night shift). This approach revealed that understaffing across the 24-hour period, particularly during the day shift (9:00 am to 5:00 pm), was associated with increased risk of in-hospital mortality, early readmission within 7 days of discharge, and longer LOS. At the time of this writing, there has been only one prior study reporting associations between shift-specific nurse understaffing and mortality, which used staffing figures at key time points (2:00 am, 10:00 am, and 6:00 pm) and found AORs of 0.73 (95% CI, 0.59-0.90) for the morning shift, 1.31 (95% CI, 1.20-1.42) for the evening shift, and 1.00 (95% CI, 0.92-1.09) for the night shift.^18^ However, these results are not directly comparable with our findings due to differences in the measurement of understaffing. In our study, the average patient-to-nurse ratio was 6:1 (4.0 NHPPD) and 5:1 (4.73 NHPPD) per 24-hour period in the understaffed and adequately staffed groups, respectively, and 3.4:1 (2.34 NHPPD) compared with 2.8:1 (2.88 NHPPD) during the day shift. Such differences in staffing suggest that adequate nurse availability during the day shift likely facilitates early detection of clinical deterioration and timely interventions, thereby reducing in-hospital mortality and LOS. Moreover, discharge planning, an essential component of nursing care that predominantly occurs during the day shift, has a substantial effect on postdischarge outcomes. Ensuring sufficient staffing during this period is critical to supporting effective discharge planning.^33^ Regular monitoring of daily staffing levels, with particular attention to the day shift, and timely responses to ward-level understaffing may contribute to improved patient outcomes.

We did not observe associations between nurse understaffing during the evening and night shift (5:00 pm to 9:00 am) and in-hospital mortality or readmissions, in contrast to some previous reports.^21,34^ Unit-level studies have identified associations between low night-shift staffing and increased adverse events,^34^ and our prior analysis indicated that older adults exposed to lower staffing levels during hospitalization relative to typical at each ward were more likely to develop hospital-associated disability.^21^ These discrepancies may reflect limitations in the measurement of certain outcomes (eg, process-of-care indicators), or they may suggest that evening and night shift staffing affects different aspects of care quality not captured by the outcomes assessed in this study. Further investigation using alternative metrics may be warranted. The lack of associations in some analyses may also reflect limited statistical power, as our sample included only 82 wards across 9 hospitals.

Sensitivity analyses suggested that maintaining nurse staffing levels at or above the current annual median, particularly during the 24-hour period or day shift and within a range of 10%, may contribute to improved patient outcomes. These results provide an actionable benchmark for nurse managers and support the ongoing monitoring and adjustment of staffing levels, as recommended in the professional judgment framework used in the UK.^35^ Given that nurse staffing levels in Japan are lower than those reported in the US, our findings offer particularly relevant evidence to inform staffing policy in settings with comparatively low staffing standards.^4,36^

### Limitations

This study has some limitations. First, the true optimal staffing level for each ward remains unknown. We used the ward-specific median as a proxy, assuming that nurse managers allocate staff according to routine, as indicated by a previous study.^35^ However, this approach may normalize chronically low staffing and limit comparability across wards. Our analysis also did not account for variations between weekdays and weekends, across specific weekdays, or during the 16-hour evening and night shifts, all of which may affect patient care needs and workload. Future research should explore data-driven approaches to estimate evidence-based, real-time staffing thresholds that incorporate such temporal heterogeneity and account for shift-specific workload. Second, although PSM was applied to reduce confounding, unmeasured variables may still have influenced the results. For instance, wards with a higher proportion of novice nurses may be more likely to maintain higher staffing levels to ensure patient safety, potentially biasing comparisons between adequately staffed and understaffed groups. Conversely, the presence of advanced practice nurses may lead to a perception that lower staffing levels are sufficient due to their ability to deliver high-quality care. These unmeasured components of nursing team composition may influence staffing decisions and patient outcomes, and they could not be fully accounted for in this study. Finally, the generalizability of our findings may be limited. Deaths within 24 hours of admission, cases with missing values (eTables 2 and 3 in Supplement 1), those more likely to involve ambulance use, and weekend admissions were excluded; including them might have overestimated the understaffing-mortality association due to lower weekend staffing. Data from NHO hospitals with more than 400 beds may not apply to smaller or private hospitals. Additionally, longer hospital stays in Japan may limit the generalizability of LOS findings to settings with shorter, more standardized hospitalization practices.

## Conclusions

This cohort study provided evidence that nurse understaffing during the 24-hour period or day shift is associated with an increased risk of in-hospital mortality, readmission, and longer LOS among patients in acute care settings. Ongoing monitoring of daily nurse staffing and addressing understaffing relative to the current annual median or higher may contribute to improved patient outcomes.

## Notes

Supplement 1. **eTable 1.** Details of the Variables **eTable 2.** Characteristics of Individuals by Death Within 24 Hours From Admission or Not **eTable 3.** Characteristics of Individuals Between the Eligible and Missing Samples **eTable 4.** Characteristics of Individuals Before and After Propensity Score Matching for 24-Hour Period Understaffing Using the Annual Median as the Cutoff Point **eTable 5.** Characteristics of Individuals Before and After Propensity Score Matching for 24-Hour Period Understaffing Using the Annual Median Plus 15% as the Cutoff **eTable 6.** Characteristics of Individuals Before and After Propensity Score Matching for 24-Hour Period Understaffing Using the Annual Median Plus 10% as the Cutoff **eTable 7.** Characteristics of Individuals Before and After Propensity Score Matching for 24-Hour Period Understaffing Using the Annual Median Plus 5% as the Cutoff **eTable 8.** Characteristics of Individuals Before and After Propensity Score Matching for 24-Hour Period Understaffing Using the Annual Median Minus 5% as the Cutoff **eTable 9.** Characteristics of Individuals Before and After Propensity Score Matching for 24-Hour Period Understaffing Using the Annual Median Minus 10% as the Cutoff **eTable 10.** Characteristics of Individuals Before and After Propensity Score Matching for 24-Hour Period Understaffing Using the Annual Median Minus 15% as the Cutoff **eTable 11.** Sensitivity Analysis for In-Hospital Death, Readmission, and Length of Stay Using Propensity Score Matching With Planned Repeated Readmission Cases **eTable 12.** Sensitivity Analysis for In-Hospital Death, Readmission, and Length of Stay Using Multilevel Models With Planned Repeated Readmission Cases **eTable 13.** Sensitivity Analysis for Length of Stay Using Propensity Score Matching With and Without In-Hospital Deaths **eTable 14.** Sensitivity Analysis for Length of Stay Using Multilevel Models With and Without In-Hospital Deaths **eTable 15.** Sensitivity Analysis for In-Hospital Death, Readmission, and Length of Stay Using Propensity Score Matching With or Without 15% Variation Around the Annual Median Cutoff **eTable 16.** Sensitivity Analysis for In-Hospital Death, Readmission, and Length of Stay Using Multilevel Models With and Without 15% Variation Around the Annual Median Cutoff **eFigure 1.** Flowchart of Sample Selection **eFigure 2.** Nurse Staffing Levels for the 24-Hour Period, Day Shift, and Evening and Night Shift

Supplement 2.
Data Sharing Statement
