# Supplementary material for: Hospital Nurse Understaffing and Patient Mortality, Readmission, and Length of Stay
Source: JAMA Netw Open. 2026 Feb 25;9(2):e2558235. doi: 10.1001/jamanetworkopen.2025.58235 (PMC12936879; doi:10.1001/jamanetworkopen.2025.58235)
Supplement: Supplement 1. — eTable 1. Details of the Variables eTable 2. Characteristics of Individuals by Death Within 24 Hours From Admission or Not eTable 3. Characteristics of Individuals Between the Eligible and Missing Samples eTable 4. Characteristics of Individuals Before and After Propensity Score Matching for 24-Hour Period Understaffing Using the Annual Median as the Cutoff Point eTable 5. Characteristics of Individuals Before and After Propensity Score Matching for 24-Hour Period Understaffing Using the Annual Median Plus 15% as the Cutoff eTable 6. Characteristics of Individuals Before and After Propensity Score Matching for 24-Hour Period Understaffing Using the Annual Median Plus 10% as the Cutoff eTable 7. Characteristics of Individuals Before and After Propensity Score Matching for 24-Hour Period Understaffing Using the Annual Median Plus 5% as the Cutoff eTable 8. Characteristics of Individuals Before and After Propensity Score Matching for 24-Hour Period Understaffing Using the Annual Median Minus 5% as the Cutoff eTable 9. Characteristics of Individuals Before and After Propensity Score Matching for 24-Hour Period Understaffing Using the Annual Median Minus 10% as the Cutoff eTable 10. Characteristics of Individuals Before and After Propensity Score Matching for 24-Hour Period Understaffing Using the Annual Median Minus 15% as the Cutoff eTable 11. Sensitivity Analysis for In-Hospital Death, Readmission, and Length of Stay Using Propensity Score Matching With Planned Repeated Readmission Cases eTable 12. Sensitivity Analysis for In-Hospital Death, Readmission, and Length of Stay Using Multilevel Models With Planned Repeated Readmission Cases eTable 13. Sensitivity Analysis for Length of Stay Using Propensity Score Matching With and Without In-Hospital Deaths eTable 14. Sensitivity Analysis for Length of Stay Using Multilevel Models With and Without In-Hospital Deaths eTable 15. Sensitivity Analysis for In-Hospital Death, Readmission, and Length of Stay Using Propensity Scor [file jamanetwopen-e2558235-s001.pdf]

Supplemental Online Content

Morioka N, Moriwaki M, Miyawaki A, Saville C, Fushimi K, Griffiths P. Hospital nurse understaffing and patient mortality, readmission, and length of stay. *JAMA Netw Open*. 2026;9(2):e2558235. doi:10.1001/jamanetworkopen.2025.58235

eTable 1. Details of the Variables

eTable 2. Characteristics of Individuals by Death Within 24 Hours From Admission or Not

eTable 3. Characteristics of Individuals Between the Eligible and Missing Samples

eTable 4. Characteristics of Individuals Before and After Propensity Score Matching for 24-Hour Period Understaffing Using the Annual Median as the Cutoff Point

eTable 5. Characteristics of Individuals Before and After Propensity Score Matching for 24-Hour Period Understaffing Using the Annual Median Plus 15% as the Cutoff

eTable 6. Characteristics of Individuals Before and After Propensity Score Matching for 24-Hour Period Understaffing Using the Annual Median Plus 10% as the Cutoff

eTable 7. Characteristics of Individuals Before and After Propensity Score Matching for 24-Hour Period Understaffing Using the Annual Median Plus 5% as the Cutoff

eTable 8. Characteristics of Individuals Before and After Propensity Score Matching for 24-Hour Period Understaffing Using the Annual Median Minus 5% as the Cutoff

eTable 9. Characteristics of Individuals Before and After Propensity Score Matching for 24-Hour Period Understaffing Using the Annual Median Minus 10% as the Cutoff

eTable 10. Characteristics of Individuals Before and After Propensity Score Matching for 24-Hour Period Understaffing Using the Annual Median Minus 15% as the Cutoff

eTable 11. Sensitivity Analysis for In-Hospital Death, Readmission, and Length of Stay Using Propensity Score Matching With Planned Repeated Readmission Cases

eTable 12. Sensitivity Analysis for In-Hospital Death, Readmission, and Length of Stay Using Multilevel Models With Planned Repeated Readmission Cases

eTable 13. Sensitivity Analysis for Length of Stay Using Propensity Score Matching With and Without In-Hospital Deaths

eTable 14. Sensitivity Analysis for Length of Stay Using Multilevel Models With and Without In-Hospital Deaths

eTable 15. Sensitivity Analysis for In-Hospital Death, Readmission, and Length of Stay Using Propensity Score Matching With or Without 15% Variation Around the Annual Median Cutoff

eTable 16. Sensitivity Analysis for In-Hospital Death, Readmission, and Length of Stay Using Multilevel Models With and Without 15% Variation Around the Annual Median Cutoff

eFigure 1. Flowchart of Sample Selection

eFigure 2. Nurse Staffing Levels for the 24-Hour Period, Day Shift, and Evening and Night Shift

This supplemental material has been provided by the authors to give readers additional information about their work.

© 2026 Morioka N et al. *JAMA Network Open*.

eTable 1. Details of the Variables

Variables	Details	Data sources
**Individual-level variables**		
Age	Years	DPC data
Sex	Male or Female	
BMI	—	
Smoking status	Brinkman Index: 0, 1–399, 400–599, 600-	
Charlson Comorbidity Index (CCI)	0, 1, 2, or ≥3	
Unconsciousness at admission	Unconscious: Japan Coma Scale (JCS) 100, 200, or 300 (=1), Conscious: JCS 0, 1, 2, 3, 10, 20, or 30 (=0)	
Ambulance use at admission	Ambulance use (=1), Not used (=0)	
Major diagnostic categories (MDCs)	18 categories:<br>1. Nervous system<br>2. Eye<br>3. Ear, nose, mouth, and throat<br>4. Respiratory system<br>5. Circulatory system<br>6. Digestive, hepatobiliary system, and pancreas<br>7. Musculoskeletal system and connective tissue<br>8. Skin and subcutaneous tissue<br>9. Breast<br>10. Endocrine, nutritional, and metabolic system<br>11. Kidney, urinary tract, and male reproductive system<br>12. Female reproductive system, pregnancy, childbirth, and puerperium<br>13. Blood, blood-forming organs, and myeloproliferative disorders<br>14. Neonatal diseases and disorders<br>15. Pediatric diseases and disorders<br>16. Trauma, burns, and poisoning<br>17. Mental diseases and disorders<br>18. Other diseases and disorders	
Weekend admission	Weekend (=1), Weekday (=0)	
ICU stay during hospitalization	ICU stay (=1), No ICU stay (=0)	
Surgery	Surgery during hospitalization (=1), No surgery (=0)	
Prehospital residence	Home, hospital/clinic, or long-term care facility	
Post-discharge residence	Home, hospital/clinic, or long-term care facility	
Severity of a Patient's Condition and Extent of a Patient's Need for Medical/Nursing Care	Three domains:<br>Item A: Intensive monitoring/treatments (7 items) (0, 1, 2, 3, or ≥4)<br>Item B: Functional status and ADLs (7 items) (0, 1, 2, 3, or ≥4)<br>Item C: Medical interventions such as surgery or emergency care (7 items) (0, 1, or ≥2)	
**Ward-level variables**		
Average number of inpatients during the hospitalization	Average number of inpatients during the hospitalization (person)	DPC data+Nurse roster data (Form No.9)
Average patient age during the hospitalization	Average patient age during the hospitalization (years)	
The average proportion of patients meeting criteria for high-acuity care	The average proportion of patients meeting criteria for high-acuity care defined by the severity score used for high-care unit classification by the Severity of a Patient's Condition and Extent of a Patient's Need for Medical/Nursing Care (%)	

© 2026 Morioka N et al. *JAMA Network Open*.

eTable 2. Characteristics of Individuals by Death Within 24 Hours From Admission or Not

	By death within 24 hours from admission or not			P-value[a]
	Not death within 24 hours from admission (eligible sample)	Death within 24 hours from admission		
		Death in the general ward	Death in emergency room	
	(N=77,289)	(N=607)	(N=906)	
Sex, n,%				
male	44,288 (57.3%)	330 (54.4%)	520 (57.4%)	0.03
female	33,001 (42.7%)	277 (45.6%)	386 (42.6%)	
Age (years), mean, SD	69.3 (15.1)	77.1 (14.0)	76.0 (14.6)	<0.001
BMI, mean, SD	23.1 (4.2)	20.7 (5.1)	21.8 (4.8)	<0.001
CCI, n,%				
0	37,806 (48.9%)	433 (71.3%)	809 (89.3%)	<0.001
1	6,009 (7.8%)	22 (3.6%)	15 (1.7%)	
2	22,629 (29.3%)	94 (15.5%)	55 (6.1%)	
3 or over	10,845 (14.0%)	58 (9.6%)	27 (3.0%)	
Smoking status (Brinkman Index), n,%				
0	39,814 (51.5%)	232 (38.2%)	209 (23.1%)	<0.001
1-399	8,235 (10.7%)	9 (1.5%)	14 (1.5%)	
400-599	4,823 (6.2%)	17 (2.8%)	4 (0.4%)	
600-	24,417 (31.6%)	349 (57.5%)	679 (74.9%)	
Prehospital residence, n,%				
Home	73,477 (95.1%)	492 (81.1%)	800 (88.4%)	<0.001
Hospital or clinic	2,092 (2.7%)	36 (5.9%)	8 (0.9%)	
Long-term care	1,720 (2.2%)	79 (13.0%)	97 (10.7%)	
Unconsciousness at admission	7,726 (10.0%)	545 (89.8%)	901 (99.4%)	<0.001
Weekend admission	9,324 (12.1%)	161 (26.5%)	245 (27.0%)	<0.001
Surgery	41,189 (53.3%)	76 (12.5%)	47 (5.2%)	<0.001
Ambulance use at admission	13,565 (17.6%)	563 (92.8%)	889 (98.1%)	<0.001
Major diagnostic categories (MDCs) , n,%				
Diseases and Disorders of the Nervous System	6,131 (7.9%)	111 (18.3%)	11 (1.2%)	<0.001
Diseases and Disorders of the Eye	5,057 (6.5%)	0 (0.0%)	0 (0.0%)	
Diseases and Disorders of the Ear, Nose, Mouth and Throat	3,026 (3.9%)	1 (0.2%)	0 (0.0%)	
Diseases and Disorders of the Respiratory System	6,909 (8.9%)	67 (11.0%)	28 (3.1%)	
Diseases and Disorders of the Circulatory System	13,228 (17.1%)	259 (42.7%)	825 (91.1%)	
Diseases and Disorders of the Digestive System, Hepatobiliary System, and Pancreas	16,757 (21.7%)	72 (11.9%)	5 (0.6%)	
Diseases and Disorders of the Musculoskeletal System and Connective Tissues	5,149 (6.7%)	1 (0.2%)	0 (0.0%)	
Diseases and Disorders of the Skin and Subcutaneous Tissue	1,545 (2.0%)	0 (0.0%)	0 (0.0%)	
Diseases and Disorders of the Breast	815 (1.1%)	3 (0.5%)	1 (0.1%)	
Diseases and Disorders of the Endocrine, Nutritional and Metabolic System	2,604 (3.4%)	5 (0.8%)	2 (0.2%)	
Diseases and Disorders of the Kidney, Urinary Tract and Male Reproductive System	7,417 (9.6%)	8 (1.3%)	2 (0.2%)	
Diseases and Disorders Pertaining to the Female Reproductive System, Pregnancy, Childbirth, and Puerperium	1,998 (2.6%)	2 (0.3%)	0 (0.0%)	
Diseases and Disorders of the Blood, Blood Forming Organ and Myeloproliferative Diseases and Disorders	1,714 (2.2%)	5 (0.8%)	1 (0.1%)	
Trauma, Burns, and Poisonings	3,403 (4.4%)	41 (6.8%)	29 (3.2%)	
Mental Diseases and Disorders	95 (0.1%)	0 (0.0%)	0 (0.0%)	
Other Diseases and Disorders	1,299 (1.7%)	32 (5.3%)	2 (0.2%)	

[a] Chi-square test or t-test

© 2026 Morioka N et al. *JAMA Network Open*.

eTable 3. Characteristics of Individuals Between the Eligible and Missing Samples

	Eligile sample (n=77,289)	Missing (n=17,448)	P-value[a]
In-hospital death	2,201 (2.8%)	941 (5.4%)	<0.001
	(n=75,088)	(n=16,507)	
7-day readmission	1,623 (2.2%)	392 (2.4%)	0.091
30-day readmission	8,160 (10.9%)	1,532 (9.3%)	<0.001
LOS	13.6 (16.1)	12.2 (16.2)	<0.001
Sex, n,%			
male	44,288 (57.3%)	6,616 (37.9%)	<0.001
female	33,001 (42.7%)	10,832 (62.1%)	
Age (years), mean, SD	69.3 (15.1)	62.3 (20.0)	<0.001
BMI, mean, SD	23.1 (4.2)	22.8 (4.8)	<0.001
CCI, n,%			
0	37,806 (48.9%)	10,965 (62.8%)	<0.001
1	6,009 (7.8%)	1,015 (5.8%)	
2	22,629 (29.3%)	3,973 (22.8%)	
3 or over	10,845 (14.0%)	1,495 (8.6%)	
Smoking status (Brinkman Index), n,%			
0	39,814 (51.5%)	10,855 (62.2%)	<0.001
1-399	8,235 (10.7%)	1,823 (10.4%)	
400-599	4,823 (6.2%)	837 (4.8%)	
600-	24,417 (31.6%)	3,933 (22.5%)	
Prehospital residence, n,%			
Home	73,477 (95.1%)	15,591 (90.6%)	<0.001
Hospital or clinic	2,092 (2.7%)	921 (5.4%)	
Long-term care	1,720 (2.2%)	692 (4.0%)	
Unconsciousness at admission	7,726 (10.0%)	3,290 (18.9%)	<0.001
Weekend admission	9,324 (12.1%)	2,507 (14.4%)	<0.001
Surgery	41,189 (53.3%)	7,096 (40.7%)	<0.001
Ambulance use at admission	13,565 (17.6%)	4,918 (28.4%)	<0.001
ICU stay during hospitalization	2,089 (2.7%)	101 (0.6%)	<0.001
Item A_Monitoring and treatment			
0	58,515 (75.7%)	15,102 (86.6%)	<0.001
1	7,134 (9.2%)	407 (2.3%)	
2	6,007 (7.8%)	546 (3.1%)	
3 or over	5,633 (7.3%)	1,393 (8.0%)	
Item B_Patients' functional status			
0	47,660 (61.7%)	14,305 (82.0%)	<0.001
1	8,148 (10.5%)	594 (3.4%)	
2	4,594 (5.9%)	358 (2.1%)	
3 or over	16,887 (21.8%)	2,191 (12.6%)	
Item C_Medical management, such as medical treatment related to surgery and emergency care			
0	75,376 (97.5%)	16,257 (93.2%)	<0.001
1 or over	1,913 (2.5%)	1,191 (6.8%)	
Major diagnostic categories (MDCs) , n,%			
Diseases and Disorders of the Nervous System	6,131 (7.9%)	1,666 (9.7%)	<0.001
Diseases and Disorders of the Eye	5,057 (6.5%)	691 (4.0%)	
Diseases and Disorders of the Ear, Nose, Mouth and Throat	3,026 (3.9%)	547 (3.2%)	
Diseases and Disorders of the Respiratory System	6,909 (8.9%)	1,823 (10.6%)	

© 2026 Morioka N et al. *JAMA Network Open*.

Diseases and Disorders of the Circulatory System	13,228 (17.1%)	1,517 (8.9%)
Diseases and Disorders of the Digestive System, Hepatobiliary System, and Pancreas	16,757 (21.7%)	2,306 (13.5%)
Diseases and Disorders of the Musculoskeletal System and Connective Tissues	5,149 (6.7%)	584 (3.4%)
Diseases and Disorders of the Skin and Subcutaneous Tissue	1,545 (2.0%)	193 (1.1%)
Diseases and Disorders of the Breast	815 (1.1%)	284 (1.7%)
Diseases and Disorders of the Endocrine, Nutritional and Metabolic System	2,604 (3.4%)	734 (4.3%)
Diseases and Disorders Pertaining to the Female Reproductive System, Pregnancy, Childbirth, and Puerperium	1,998 (2.6%)	3,531 (20.6%)
Diseases and Disorders of the Blood, Blood Forming Organ and Myeloproliferative Diseases and Disorders	1,714 (2.2%)	126 (0.7%)
Pediatric Diseases and Disorder	142 (0.2%)	20 (0.1%)
Trauma, Burns, and Poisonings	3,403 (4.4%)	1,403 (8.2%)
Mental Diseases and Disorders	95 (0.1%)	682 (4.0%)
Other Diseases and Disorders	1,299 (1.7%)	223 (1.3%)

[a] Chi-square test or t-test

© 2026 Morioka N et al. *JAMA Network Open*.

eTable 4. Characteristics of Individuals Before and After Propensity Score Matching for 24-Hour Period Understaffing Using the Annual Median as the Cutoff Point

	Before PS matching			After PS[a] matching for in-hospital death and LOS			After PS[a] matching for readmission		
	Adequately staffed group (>=annual median) (N=43,770)	Understaffed group (below annual median) (N=33,516)	P-value[b]	Adequately staffed group (>=annual median) (N=28,846)	Understaffed group (below annual median) (N=28,846)	P-value[b]	Adequately staffed group (>=annual median) (N=27,907)	Understaffed group (below annual median) (N=27,907)	P-value[b]
NHPPD	4.73 (0.84)	4.00 (0.66)	<0.001	4.53 (0.72)	4.05 (0.68)	<0.001	4.54 (0.72)	4.06 (0.68)	<0.001
Sex, n,%									
female	18,599 (42.5%)	14,476 (43.2%)	0.052	12,396 (43.0%)	12,161 (42.2%)	0.048	12,092 (43.3%)	11,806 (42.3%)	0.014
Age (years), mean, SD	69.12 (15.00)	69.44 (15.28)	0.003	69.41 (15.18)	69.31 (15.01)	0.428	69.12 (15.22)	69.10 (15.00)	0.876
BMI, mean, SD	23.13 (4.14)	23.05 (4.20)	0.015	23.05 (4.16)	23.10 (4.21)	0.176	23.15 (4.15)	23.17 (4.19)	0.536
CCI, n,%									
0	21,795 (49.8%)	15,992 (47.7%)	<0.001	13,848 (48.0%)	13,917 (48.2%)	0.766	13,660 (48.9%)	13,677 (49.0%)	0.915
1	3,378 (7.7%)	2,654 (7.9%)		2,294 (8.0%)	2,259 (7.8%)		2,237 (8.0%)	2,223 (8.0%)	
2	12,593 (28.8%)	10,017 (29.9%)		8,504 (29.5%)	8,542 (29.6%)		8,190 (29.3%)	8,237 (29.5%)	
3 or over	6,004 (13.7%)	4,853 (14.5%)		4,200 (14.6%)	4,128 (14.3%)		3,820 (13.7%)	3,770 (13.5%)	
Smoking status (Brinkman Index), n,%									
0	22,402 (51.2%)	17,413 (52.0%)	0.063	14,942 (51.8%)	14,778 (51.2%)	0.589	14,476 (51.9%)	14,322 (51.3%)	0.476
1-399	4,716 (10.8%)	3,515 (10.5%)		3,026 (10.5%)	3,078 (10.7%)		2,950 (10.6%)	3,022 (10.8%)	
400-599	2,694 (6.2%)	2,123 (6.3%)		1,792 (6.2%)	1,811 (6.3%)		1,768 (6.3%)	1,744 (6.2%)	
600-	13,958 (31.9%)	10,465 (31.2%)		9,086 (31.5%)	9,179 (31.8%)		8,713 (31.2%)	8,819 (31.6%)	
Prehospital residence, n,%									
Home	41,763 (95.4%)	31,714 (94.6%)	<0.001	27,348 (94.8%)	27,398 (95.0%)	0.508	26,525 (95.0%)	26,577 (95.2%)	0.587
Hospital or clinic	1,112 (2.5%)	977 (2.9%)		810 (2.8%)	801 (2.8%)		758 (2.7%)	733 (2.6%)	
Long-term care	895 (2.0%)	825 (2.5%)		688 (2.4%)	647 (2.2%)		624 (2.2%)	597 (2.1%)	
Unconsciousness at admission	3,923 (9.0%)	3,798 (11.3%)	<0.001	2,993 (10.4%)	2,831 (9.8%)	0.025	2,712 (9.7%)	2,538 (9.1%)	0.012
Weekend admission	4,991 (11.4%)	4,329 (12.9%)	<0.001	3,580 (12.4%)	3,390 (11.8%)	0.015	3,408 (12.2%)	3,228 (11.6%)	0.019
Surgery	23,054 (52.7%)	18,083 (54.0%)	<0.001	15,448 (53.6%)	15,421 (53.5%)	0.822	15,208 (54.5%)	15,163 (54.3%)	0.702
Ambulance use at admission	6,917 (15.8%)	6,653 (19.9%)	<0.001	5,344 (18.5%)	4,877 (16.9%)	<0.001	4,954 (17.8%)	4,543 (16.3%)	<0.001
ICU stay during hospitalization	1,122 (2.6%)	974 (2.9%)	0.004	804 (2.8%)	838 (2.9%)	0.395	808 (2.9%)	809 (2.9%)	0.98
Major diagnostic categories (MDCs) , n,%									
Diseases and Disorders of the Nervous System	3,361 (7.7%)	2,776 (8.3%)	<0.001	2,294 (8.0%)	2,364 (8.2%)	0.029	2,196 (7.9%)	2,290 (8.2%)	0.057
Diseases and Disorders of the Eye	3,252 (7.4%)	1,773 (5.3%)		1,642 (5.7%)	1,704 (5.9%)		1,646 (5.9%)	1,692 (6.1%)	
Diseases and Disorders of the Ear, Nose, Mouth and Throat	1,695 (3.9%)	1,335 (4.0%)		1,144 (4.0%)	1,144 (4.0%)		1,149 (4.1%)	1,112 (4.0%)	
Diseases and Disorders of the Respiratory System	3,769 (8.6%)	3,145 (9.4%)		2,726 (9.5%)	2,640 (9.2%)		2,518 (9.0%)	2,423 (8.7%)	
Diseases and Disorders of the Circulatory System	7,694 (17.6%)	5,548 (16.6%)		4,605 (16.0%)	4,902 (17.0%)		4,608 (16.5%)	4,815 (17.3%)	
System, Hepatobiliary System, and Pancreas	9,394 (21.5%)	7,294 (21.8%)		6,424 (22.3%)	6,402 (22.2%)		6,086 (21.8%)	6,129 (22.0%)	
Diseases and Disorders of the Musculoskeletal System and Connective Tissues	2,872 (6.6%)	2,286 (6.8%)		1,982 (6.9%)	1,998 (6.9%)		1,987 (7.1%)	1,980 (7.1%)	
Diseases and Disorders of the Skin and Subcutaneous Tissue	861 (2.0%)	685 (2.0%)		583 (2.0%)	564 (2.0%)		580 (2.1%)	566 (2.0%)	
Diseases and Disorders of the Breast	449 (1.0%)	367 (1.1%)		319 (1.1%)	322 (1.1%)		302 (1.1%)	312 (1.1%)	
Diseases and Disorders of the Endocrine, Nutritional and Metabolic System	1,426 (3.3%)	1,181 (3.5%)		1,030 (3.6%)	957 (3.3%)		1,003 (3.6%)	946 (3.4%)	
Diseases and Disorders of the Kidney, Urinary Tract and Male Reproductive System	4,243 (9.7%)	3,197 (9.5%)		2,816 (9.8%)	2,814 (9.8%)		2,699 (9.7%)	2,753 (9.9%)	
Diseases and Disorders Pertaining to the Female Reproductive System, Pregnancy, Childbirth, and Puerperium	1,129 (2.6%)	897 (2.7%)		685 (2.4%)	610 (2.1%)		691 (2.5%)	591 (2.1%)	
Diseases and Disorders of the Blood, Blood Forming Organ and Myeloproliferative Diseases and Disorders	986 (2.3%)	732 (2.2%)		662 (2.3%)	645 (2.2%)		594 (2.1%)	584 (2.1%)	
Pediatric Diseases and Disorder	82 (0.2%)	60 (0.2%)		48 (0.2%)	53 (0.2%)		58 (0.2%)	55 (0.2%)	
Trauma, Burns, and Poisonings	1,811 (4.1%)	1,593 (4.8%)		1,344 (4.7%)	1,219 (4.2%)		1,280 (4.6%)	1,195 (4.3%)	
Mental Diseases and Disorders	49 (0.1%)	41 (0.1%)		35 (0.1%)	37 (0.1%)		41 (0.1%)	32 (0.1%)	
Other Diseases and Disorders	697 (1.6%)	606 (1.8%)		507 (1.8%)	471 (1.6%)		469 (1.7%)	432 (1.5%)	
Severity of a Patient's Condition and Extent of a Patient's Need for Medical/Nursing Care index[c]									
Item A_Monitoring and treatment									
0	33,431 (76.4%)	25,211 (75.2%)	<0.001	21,800 (75.6%)	21,941 (76.1%)	0.273	21,207 (76.0%)	21,390 (76.6%)	0.172
1	4,107 (9.4%)	3,035 (9.1%)		2,642 (9.2%)	2,649 (9.2%)		2,591 (9.3%)	2,568 (9.2%)	
2	3,252 (7.4%)	2,769 (8.3%)		2,338 (8.1%)	2,216 (7.7%)		2,223 (8.0%)	2,094 (7.5%)	
3 or over	2,980 (6.8%)	2,501 (7.5%)		2,066 (7.2%)	2,040 (7.1%)		1,886 (6.8%)	1,855 (6.6%)	
Item B_Patients' functional status									
0	27,195 (62.1%)	20,573 (61.4%)	<0.001	17,590 (61.0%)	17,856 (61.9%)	0.088	17,261 (61.9%)	17,496 (62.7%)	0.067
1	4,752 (10.9%)	3,414 (10.2%)		3,043 (10.5%)	3,034 (10.5%)		2,962 (10.6%)	2,984 (10.7%)	
2	2,696 (6.2%)	1,908 (5.7%)		1,714 (5.9%)	1,695 (5.9%)		1,641 (5.9%)	1,639 (5.9%)	
3 or over	9,127 (20.9%)	7,621 (22.7%)		6,499 (22.5%)	6,261 (21.7%)		6,043 (21.7%)	5,788 (20.7%)	
Item C_Medical management, such as medical treatment related to surgery and emergency care									
0	42,793 (97.8%)	32,733 (97.7%)	0.336	28,178 (97.7%)	28,174 (97.7%)	0.912	27,263 (97.7%)	27,247 (97.6%)	0.654
1 or over	977 (2.2%)	783 (2.3%)		668 (2.3%)	672 (2.3%)		644 (2.3%)	660 (2.4%)	

© 2026 Morioka N et al. *JAMA Network Open*.

**Ward level variables**									
Average number of inpatients during the hospitalization, mean, SD	37.06 (6.44)	39.56 (6.05)	<0.001	38.82 (5.62)	38.92 (6.17)	0.043	38.80 (5.61)	38.91 (6.15)	0.036
Aaverage patient age during the hospitalization (years), mean, SD	71.16 (3.43)	71.37 (3.52)	<0.001	71.31 (3.30)	71.30 (3.49)	0.768	71.30 (3.33)	71.28 (3.49)	0.446
meeting criteria for high-acuity caren (%), mean, SD	21.63 (10.27)	20.66 (9.42)	<0.001	20.68 (9.64)	21.22 (9.49)	<0.001	20.67 (9.68)	21.22 (9.50)	<0.001

[a] In the propensity score estimation, adjustments were made for age, age squared, Charlson Comorbidity Index (CCI), smoking index, body mass index (BMI), place of residence before admission, ICU stay, unconsciousness at admission, ambulance use, weekend admission, place after discharge (for the readmission model only), main diagnosis category, the Japanese Severity and Medical/Nursing Care Needs Score (Items A, B, and C) at admission, percentage of severe inpatients in the ward, median age of inpatients in the ward, number of inpatients in the ward, and hospital fixed effects (dummy variables).

[b] Chi-square test or t-test

[c] The Severity of a Patient's Condition and Extent of a Patient's Need for Medical/Nursing Care for General Wards comprises 21 items. Item A (7 items) assesses the need for specialized nursing care, including monitoring and treatment; Item B (7 items) evaluates patients' functional status, such as activities of daily living (ADLs), that influence health service use; and Item C (7 items) measures the need for medical management, including treatments related to surgery and emergency care.

© 2026 Morioka N et al. *JAMA Network Open*.

eTable 5. Characteristics of Individuals Before and After Propensity Score Matching for 24-Hour Period Understaffing Using the Annual Median Plus 15% as the Cutoff

	Before PS matching			After PS[a] matching for in-hospital death and LOS			After PS[a] matching for readmission		
	Adequately staffed group (>=annual median+15%) (N=10,382)	Understaffed group (below annual median+15%) (N=66,904)	P-value[b]	Adequately staffed group (>=annual median+15%) (N=10,379)	Understaffed group (below annual median+15%) (N=10,379)	P-value[b]	Adequately staffed group (>=annual median+15%) (N=10,170)	Understaffed group (below annual median+15%) (N=10,170)	P-value[b]
NHPPD	5.3 (0.8)	4.3 (0.8)	<0.001	5.32 (0.83)	4.84 (1.00)	<0.001	5.33 (0.83)	4.86 (1.00)	<0.001
Sex, n,%	6,008 (57.9%)	38,203 (57.1%)	0.141	6,005 (57.9%)	6,004 (57.8%)	0.989	5,882 (57.8%)	5,883 (57.8%)	0.989
female	4,374 (42.1%)	28,701 (42.9%)		4,374 (42.1%)	4,375 (42.2%)		4,288 (42.2%)	4,287 (42.2%)	
Age (years), mean, SD	68.7 (14.8)	69.3 (15.2)	<0.001	68.70 (14.79)	68.27 (15.12)	0.041	68.51 (14.78)	68.02 (15.07)	0.018
BMI, mean, SD	23.2 (4.1)	23.1 (4.2)	<0.001	23.24 (4.09)	23.22 (4.20)	0.677	23.30 (4.07)	23.28 (4.19)	0.702
CCI, n,%									
0	5,453 (52.5%)	32,334 (48.3%)	<0.001	5,450 (52.5%)	5,498 (53.0%)	0.711	5,407 (53.2%)	5,441 (53.5%)	0.926
1	712 (6.9%)	5,320 (8.0%)		712 (6.9%)	672 (6.5%)		701 (6.9%)	691 (6.8%)	
2	2,940 (28.3%)	19,670 (29.4%)		2,940 (28.3%)	2,932 (28.2%)		2,874 (28.3%)	2,876 (28.3%)	
3 or over	1,277 (12.3%)	9,580 (14.3%)		1,277 (12.3%)	1,277 (12.3%)		1,188 (11.7%)	1,162 (11.4%)	
Smoking status (Brinkman Index), n,%									
0	5,231 (50.4%)	34,584 (51.7%)	0.002	5,229 (50.4%)	5,157 (49.7%)	0.746	5,115 (50.3%)	5,006 (49.2%)	0.407
1-399	1,187 (11.4%)	7,044 (10.5%)		1,187 (11.4%)	1,183 (11.4%)		1,176 (11.6%)	1,172 (11.5%)	
400-599	700 (6.7%)	4,117 (6.2%)		700 (6.7%)	712 (6.9%)		683 (6.7%)	692 (6.8%)	
600-	3,264 (31.4%)	21,159 (31.6%)		3,263 (31.4%)	3,327 (32.1%)		3,196 (31.4%)	3,300 (32.4%)	
Prehospital residence, n,%									
Home	10,041 (96.7%)	63,436 (94.8%)	<0.001	10,038 (96.7%)	10,071 (97.0%)	0.117	9,860 (97.0%)	9,896 (97.3%)	0.180
Hospital or clinic	182 (1.8%)	1,907 (2.9%)		182 (1.8%)	145 (1.4%)		171 (1.7%)	139 (1.4%)	
Long-term care	159 (1.5%)	1,561 (2.3%)		159 (1.5%)	163 (1.6%)		139 (1.4%)	135 (1.3%)	
Unconsciousness at admission	769 (7.4%)	6,952 (10.4%)	<0.001	769 (7.4%)	722 (7.0%)	0.206	692 (6.8%)	601 (5.9%)	0.009
Weekend admission	1,026 (9.9%)	8,294 (12.4%)	<0.001	1,026 (9.9%)	1,027 (9.9%)	0.981	992 (9.8%)	989 (9.7%)	0.943
Surgery	5,205 (50.1%)	35,932 (53.7%)	<0.001	5,203 (50.1%)	5,004 (48.2%)	0.006	5,162 (50.8%)	5,033 (49.5%)	0.070
Ambulance use at admission	1,336 (12.9%)	12,234 (18.3%)	<0.001	1,336 (12.9%)	1,253 (12.1%)	0.081	1,249 (12.3%)	1,147 (11.3%)	0.027
ICU stay during hospitalization	160 (1.5%)	1,936 (2.9%)	<0.001	160 (1.5%)	128 (1.2%)	0.058	154 (1.5%)	122 (1.2%)	0.052
Major diagnostic categories (MDCs) , n,%									
Diseases and Disorders of the Nervous System	833 (8.0%)	5,304 (7.9%)	<0.001	833 (8.0%)	792 (7.6%)	<0.001	816 (8.0%)	754 (7.4%)	<0.001
Diseases and Disorders of the Eye	1,040 (10.0%)	3,985 (6.0%)		1,040 (10.0%)	1,033 (10.0%)		1,039 (10.2%)	1,074 (10.6%)	
Diseases and Disorders of the Ear, Nose, Mouth and Throat	381 (3.7%)	2,649 (4.0%)		380 (3.7%)	351 (3.4%)		377 (3.7%)	364 (3.6%)	
Diseases and Disorders of the Respiratory System	772 (7.4%)	6,142 (9.2%)		772 (7.4%)	792 (7.6%)		722 (7.1%)	737 (7.2%)	
Diseases and Disorders of the Circulatory System	1,899 (18.3%)	11,343 (17.0%)		1,899 (18.3%)	1,893 (18.2%)		1,880 (18.5%)	1,845 (18.1%)	
System, Hepatobiliary System, and Pancreas	2,398 (23.1%)	14,290 (21.4%)		2,396 (23.1%)	2,508 (24.2%)		2,326 (22.9%)	2,456 (24.1%)	
Diseases and Disorders of the Musculoskeletal System and Connective Tissues	464 (4.5%)	4,694 (7.0%)		464 (4.5%)	374 (3.6%)		462 (4.5%)	383 (3.8%)	
Diseases and Disorders of the Skin and Subcutaneous Tissue	245 (2.4%)	1,301 (1.9%)		245 (2.4%)	272 (2.6%)		244 (2.4%)	268 (2.6%)	
Diseases and Disorders of the Breast	128 (1.2%)	688 (1.0%)		128 (1.2%)	108 (1.0%)		124 (1.2%)	110 (1.1%)	
Diseases and Disorders of the Endocrine, Nutritional and Metabolic System	275 (2.6%)	2,332 (3.5%)		275 (2.6%)	250 (2.4%)		274 (2.7%)	232 (2.3%)	
Diseases and Disorders of the Kidney, Urinary Tract and Male Reproductive System	933 (9.0%)	6,507 (9.7%)		933 (9.0%)	926 (8.9%)		918 (9.0%)	879 (8.6%)	
Diseases and Disorders Pertaining to the Female Reproductive System, Pregnancy, Childbirth, and Puerperium	351 (3.4%)	1,675 (2.5%)		351 (3.4%)	480 (4.6%)		350 (3.4%)	483 (4.7%)	
Diseases and Disorders of the Blood, Blood Forming Organ and Myeloproliferative Diseases and Disorders	186 (1.8%)	1,532 (2.3%)		186 (1.8%)	179 (1.7%)		176 (1.7%)	181 (1.8%)	
Pediatric Diseases and Disorder	20 (0.2%)	122 (0.2%)		20 (0.2%)	15 (0.1%)		20 (0.2%)	11 (0.1%)	
Trauma, Burns, and Poisonings	284 (2.7%)	3,120 (4.7%)		284 (2.7%)	245 (2.4%)		278 (2.7%)	231 (2.3%)	
Mental Diseases and Disorders	17 (0.2%)	73 (0.1%)		17 (0.2%)	19 (0.2%)		17 (0.2%)	15 (0.1%)	
Other Diseases and Disorders	156 (1.5%)	1,147 (1.7%)		156 (1.5%)	142 (1.4%)		147 (1.4%)	147 (1.4%)	
Severity of a Patient's Condition and Extent of a Patient's Need for Medical/Nursing Care index[c]									
Item A_Monitoring and treatment									
0	8,013 (77.2%)	50,629 (75.7%)	<0.001	8,010 (77.2%)	7,917 (76.3%)	0.396	7,910 (77.8%)	7,823 (76.9%)	0.497
1	987 (9.5%)	6,155 (9.2%)		987 (9.5%)	1,051 (10.1%)		968 (9.5%)	1,010 (9.9%)	
2	704 (6.8%)	5,317 (7.9%)		704 (6.8%)	728 (7.0%)		670 (6.6%)	706 (6.9%)	
3 or over	678 (6.5%)	4,803 (7.2%)		678 (6.5%)	683 (6.6%)		622 (6.1%)	631 (6.2%)	
Item B_Patients' functional status									
0	6,487 (62.5%)	41,281 (61.7%)	<0.001	6,484 (62.5%)	6,360 (61.3%)	0.261	6,424 (63.2%)	6,298 (61.9%)	0.172
1	1,172 (11.3%)	6,994 (10.5%)		1,172 (11.3%)	1,173 (11.3%)		1,161 (11.4%)	1,166 (11.5%)	
2	686 (6.6%)	3,918 (5.9%)		686 (6.6%)	727 (7.0%)		674 (6.6%)	738 (7.3%)	
3 or over	2,037 (19.6%)	14,711 (22.0%)		2,037 (19.6%)	2,119 (20.4%)		1,911 (18.8%)	1,968 (19.4%)	
Item C_Medical management, such as medical treatment related to surgery and emergency care									
0	10,082 (97.1%)	65,444 (97.8%)	<0.001	10,079 (97.1%)	10,072 (97.0%)	0.773	9,871 (97.1%)	9,849 (96.8%)	0.370
1 or over	300 (2.9%)	1,460 (2.2%)		300 (2.9%)	307 (3.0%)		299 (2.9%)	321 (3.2%)	

© 2026 Morioka N et al. *JAMA Network Open*.

**Ward level variables**									
Average number of inpatients during the hospitalization, mean, SD	34.5 (6.4)	38.7 (6.2)	<0.001	34.54 (6.42)	33.32 (7.75)	<0.001	34.51 (6.40)	33.32 (7.69)	<0.001
Aaverage patient age during the hospitalization (years), mean, SD	71.1 (3.4)	71.3 (3.5)	<0.001	71.05 (3.35)	70.99 (3.94)	0.203	71.03 (3.35)	70.97 (3.93)	0.278
meeting criteria for high-acuity caren (%), mean, SD	22.7 (11.7)	21.0 (9.6)	<0.001	22.71 (11.66)	22.55 (10.81)	0.295	22.72 (11.69)	22.51 (10.85)	0.184

[a] In the propensity score estimation, adjustments were made for age, age squared, Charlson Comorbidity Index (CCI), smoking index, body mass index (BMI), place of residence before admission, ICU stay, unconsciousness at admission, ambulance use, weekend admission, place after discharge (for the readmission model only), main diagnosis category, the Japanese Severity and Medical/Nursing Care Needs Score (Items A, B, and C) at admission, percentage of severe inpatients in the ward, median age of inpatients in the ward, number of inpatients in the ward, and hospital fixed effects (dummy variables).

[b] Chi-square test or t-test

[c] The Severity of a Patient's Condition and Extent of a Patient's Need for Medical/Nursing Care for General Wards comprises 21 items. Item A (7 items) assesses the need for specialized nursing care, including monitoring and treatment; Item B (7 items) evaluates patients' functional status, such as activities of daily living (ADLs), that influence health service use; and Item C (7 items) measures the need for medical management, including treatments related to surgery and emergency care.

© 2026 Morioka N et al. *JAMA Network Open*.

eTable 6. Characteristics of Individuals Before and After Propensity Score Matching for 24-Hour Period Understaffing Using the Annual Median Plus 10% as the Cutoff

	Before PS matching			After PS[a] matching for in-hospital death and LOS			After PS[a] matching for readmission		
	Adequately staffed group (>=annual median +10% point) (N=17,337)	Understaffed group (belowannual median +10% point) (N=59,949)	P-value[b]	Adequately staffed group (>=annual median +10% point) (N=16,925)	Understaffed group (belowannual median +10% point) (N=16,925)	P-value[b]	Adequately staffed group (>=annual median +10% point) (N=16,526)	Understaffed group (belowannual median +10% point) (N=16,526)	P-value[b]
NHPPD	5.11 (0.86)	4.21 (0.73)	<0.001	5.09 (0.84)	4.61 (0.91)	<0.001	5.09 (0.84)	4.61 (0.91)	<0.001
Sex, n,%									
female	7,383 (42.6%)	25,692 (42.9%)	0.525	7,204 (42.6%)	7,120 (42.1%)	0.355	7,043 (42.6%)	7,027 (42.5%)	0.859
Age (years), mean, SD	68.77 (14.87)	69.40 (15.19)	<0.001	68.77 (14.89)	68.52 (14.93)	0.118	68.58 (14.90)	68.22 (14.93)	0.025
BMI, mean, SD	23.20 (4.09)	23.06 (4.19)	<0.001	23.20 (4.08)	23.24 (4.23)	0.393	23.25 (4.07)	23.28 (4.19)	0.534
CCI, n,%									
0	8,923 (51.5%)	28,864 (48.1%)	<0.001	8,692 (51.4%)	8,826 (52.1%)	0.400	8,588 (52.0%)	8,749 (52.9%)	0.254
1	1,251 (7.2%)	4,781 (8.0%)		1,233 (7.3%)	1,180 (7.0%)		1,209 (7.3%)	1,164 (7.0%)	
2	4,883 (28.2%)	17,727 (29.6%)		4,774 (28.2%)	4,748 (28.1%)		4,653 (28.2%)	4,528 (27.4%)	
3 or over	2,280 (13.2%)	8,577 (14.3%)		2,226 (13.2%)	2,171 (12.8%)		2,076 (12.6%)	2,085 (12.6%)	
Smoking status (Brinkman Index), n,%									
0	8,822 (50.9%)	30,993 (51.7%)	0.002	8,602 (50.8%)	8,506 (50.3%)	0.372	8,403 (50.8%)	8,332 (50.4%)	0.684
1-399	1,970 (11.4%)	6,261 (10.4%)		1,916 (11.3%)	1,933 (11.4%)		1,898 (11.5%)	1,925 (11.6%)	
400-599	1,117 (6.4%)	3,700 (6.2%)		1,086 (6.4%)	1,039 (6.1%)		1,054 (6.4%)	1,023 (6.2%)	
600-	5,428 (31.3%)	18,995 (31.7%)		5,321 (31.4%)	5,447 (32.2%)		5,171 (31.3%)	5,246 (31.7%)	
Prehospital residence, n,%									
Home	16,712 (96.4%)	56,765 (94.7%)	<0.001	16,308 (96.4%)	16,387 (96.8%)	0.051	15,962 (96.6%)	16,039 (97.1%)	0.048
Hospital or clinic	333 (1.9%)	1,756 (2.9%)		330 (1.9%)	278 (1.6%)		305 (1.8%)	256 (1.5%)	
Long-term care	292 (1.7%)	1,428 (2.4%)		287 (1.7%)	260 (1.5%)		259 (1.6%)	231 (1.4%)	
Unconsciousness at admission	1,377 (7.9%)	6,344 (10.6%)	<0.001	1,357 (8.0%)	1,262 (7.5%)	0.053	1,230 (7.4%)	1,130 (6.8%)	0.033
Weekend admission	1,872 (10.8%)	7,448 (12.4%)	<0.001	1,826 (10.8%)	1,780 (10.5%)	0.418	1,766 (10.7%)	1,715 (10.4%)	0.361
Surgery	8,903 (51.4%)	32,234 (53.8%)	<0.001	8,707 (51.4%)	8,614 (50.9%)	0.312	8,616 (52.1%)	8,398 (50.8%)	0.016
Ambulance use at admission	2,354 (13.6%)	11,216 (18.7%)	<0.001	2,323 (13.7%)	2,141 (12.6%)	0.003	2,172 (13.1%)	1,993 (12.1%)	0.003
ICU stay during hospitalization	294 (1.7%)	1,802 (3.0%)	<0.001	288 (1.7%)	188 (1.1%)	<0.001	277 (1.7%)	190 (1.1%)	<0.001
Major diagnostic categories (MDCs) , n,%									
Diseases and Disorders of the Nervous System	1,328 (7.7%)	4,809 (8.0%)	<0.001	1,312 (7.8%)	1,222 (7.2%)	<0.001	1,283 (7.8%)	1,208 (7.3%)	<0.001
Diseases and Disorders of the Eye	1,557 (9.0%)	3,468 (5.8%)		1,502 (8.9%)	1,612 (9.5%)		1,504 (9.1%)	1,621 (9.8%)	
Diseases and Disorders of the Ear, Nose, Mouth and Throat	658 (3.8%)	2,372 (4.0%)		646 (3.8%)	621 (3.7%)		635 (3.8%)	616 (3.7%)	
Diseases and Disorders of the Respiratory System	1,368 (7.9%)	5,546 (9.3%)		1,340 (7.9%)	1,296 (7.7%)		1,251 (7.6%)	1,196 (7.2%)	
Diseases and Disorders of the Circulatory System	3,155 (18.2%)	10,087 (16.8%)		3,083 (18.2%)	3,114 (18.4%)		3,056 (18.5%)	3,130 (18.9%)	
System, Hepatobiliary System, and Pancreas	3,882 (22.4%)	12,806 (21.4%)		3,756 (22.2%)	3,870 (22.9%)		3,628 (22.0%)	3,760 (22.8%)	
Diseases and Disorders of the Musculoskeletal System and Connective Tissues	890 (5.1%)	4,268 (7.1%)		881 (5.2%)	698 (4.1%)		870 (5.3%)	726 (4.4%)	
Diseases and Disorders of the Skin and Subcutaneous Tissue	389 (2.2%)	1,157 (1.9%)		379 (2.2%)	409 (2.4%)		370 (2.2%)	395 (2.4%)	
Diseases and Disorders of the Breast	186 (1.1%)	630 (1.1%)		183 (1.1%)	180 (1.1%)		176 (1.1%)	172 (1.0%)	
Diseases and Disorders of the Endocrine, Nutritional and Metabolic System	510 (2.9%)	2,097 (3.5%)		505 (3.0%)	430 (2.5%)		500 (3.0%)	426 (2.6%)	
Diseases and Disorders of the Kidney, Urinary Tract and Male Reproductive System	1,616 (9.3%)	5,824 (9.7%)		1,586 (9.4%)	1,666 (9.8%)		1,560 (9.4%)	1,527 (9.2%)	
Diseases and Disorders Pertaining to the Female Reproductive System, Pregnancy, Childbirth, and Puerperium	561 (3.2%)	1,465 (2.4%)		542 (3.2%)	665 (3.9%)		537 (3.2%)	653 (4.0%)	
Diseases and Disorders of the Blood, Blood Forming Organ and Myeloproliferative Diseases and Disorders	357 (2.1%)	1,361 (2.3%)		350 (2.1%)	326 (1.9%)		314 (1.9%)	329 (2.0%)	
Pediatric Diseases and Disorder	33 (0.2%)	109 (0.2%)		32 (0.2%)	37 (0.2%)		33 (0.2%)	37 (0.2%)	
Trauma, Burns, and Poisonings	565 (3.3%)	2,839 (4.7%)		554 (3.3%)	490 (2.9%)		545 (3.3%)	475 (2.9%)	
Mental Diseases and Disorders	26 (0.1%)	64 (0.1%)		25 (0.1%)	23 (0.1%)		24 (0.1%)	28 (0.2%)	
Other Diseases and Disorders	256 (1.5%)	1,047 (1.7%)		249 (1.5%)	266 (1.6%)		240 (1.5%)	227 (1.4%)	
Severity of a Patient's Condition and Extent of a Patient's Need for Medical/Nursing Care index[c]									
Item A_Monitoring and treatment									
0	13,308 (76.8%)	45,334 (75.6%)	<0.001	12,992 (76.8%)	12,903 (76.2%)	0.125	12,768 (77.3%)	12,677 (76.7%)	0.240
1	1,707 (9.8%)	5,435 (9.1%)		1,661 (9.8%)	1,790 (10.6%)		1,639 (9.9%)	1,746 (10.6%)	
2	1,202 (6.9%)	4,819 (8.0%)		1,172 (6.9%)	1,168 (6.9%)		1,116 (6.8%)	1,129 (6.8%)	
3 or over	1,120 (6.5%)	4,361 (7.3%)		1,100 (6.5%)	1,064 (6.3%)		1,003 (6.1%)	974 (5.9%)	
Item B_Patients' functional status									
0	10,839 (62.5%)	36,929 (61.6%)	<0.001	10,586 (62.5%)	10,601 (62.6%)	0.944	10,438 (63.2%)	10,361 (62.7%)	0.770
1	1,910 (11.0%)	6,256 (10.4%)		1,853 (10.9%)	1,879 (11.1%)		1,838 (11.1%)	1,887 (11.4%)	
2	1,108 (6.4%)	3,496 (5.8%)		1,075 (6.4%)	1,066 (6.3%)		1,048 (6.3%)	1,068 (6.5%)	
3 or over	3,480 (20.1%)	13,268 (22.1%)		3,411 (20.2%)	3,379 (20.0%)		3,202 (19.4%)	3,210 (19.4%)	

© 2026 Morioka N et al. *JAMA Network Open*.

Item C_Medical management, such as medical treatment related to surgery and emergency care									
0	16,889 (97.4%)	58,637 (97.8%)	0.002	16,489 (97.4%)	16,479 (97.4%)	0.733	16,093 (97.4%)	16,073 (97.3%)	0.496
1 or over	448 (2.6%)	1,312 (2.2%)		436 (2.6%)	446 (2.6%)		433 (2.6%)	453 (2.7%)	
**Ward level variables**									
Number of inpatients on a ward during hospitalization, mean, SD	35.43 (6.57)	38.93 (6.12)	<0.001	35.62 (6.49)	34.79 (7.44)	<0.001	35.62 (6.45)	34.82 (7.39)	<0.001
Median age of inpatients on a ward during hospitalization (years), mean, SD	71.06 (3.46)	71.30 (3.47)	<0.001	71.07 (3.45)	71.08 (3.75)	0.779	71.05 (3.46)	71.01 (3.76)	0.360
a ward during hospitalization (%), mean, SD	22.30 (11.08)	20.89 (9.53)	<0.001	22.13 (10.93)	22.10 (10.32)	0.833	22.14 (10.95)	22.11 (10.37)	0.814

[a] In the propensity score estimation, adjustments were made for age, age squared, Charlson Comorbidity Index (CCI), smoking index, body mass index (BMI), place of residence before admission, ICU stay, unconsciousness at admission, ambulance use, weekend admission, place after discharge (for the readmission model only), main diagnosis category, the Japanese Severity and Medical/Nursing Care Needs Score (Items A, B, and C) at admission, percentage of severe inpatients in the ward, median age of inpatients in the ward, number of inpatients in the ward, and hospital fixed effects (dummy variables).

[b] Chi-square test or t-test

[c] The Severity of a Patient's Condition and Extent of a Patient's Need for Medical/Nursing Care for General Wards comprises 21 items. Item A (7 items) assesses the need for specialized nursing care, including monitoring and treatment; Item B (7 items) evaluates patients' functional status, such as activities of daily living (ADLs), that influence health service use; and Item C (7 items) measures the need for medical management, including treatments related to surgery and emergency care.

© 2026 Morioka N et al. *JAMA Network Open*.

eTable 7. Characteristics of Individuals Before and After Propensity Score Matching for 24-Hour Period Understaffing Using the Annual Median Plus 5% as the Cutoff

	Before PS matching			After PS[a] matching for in-hospital death and LOS			After PS[a] matching for readmission		
	Adequately staffed group (>=annual median +5% point) (N=28,556)	Understaffed group (belowannual median +5% point) (N=48,730)	P-value[b]	Adequately staffed group (>=annual median +5% point) (N=25,770)	Understaffed group (belowannual median +5% point) (N=25,770)	P-value[b]	Adequately staffed group (>=annual median +5% point) (N=25,061)	Understaffed group (belowannual median +5% point) (N=25,061)	P-value[b]
NHPPD	4.91 (0.85)	4.12 (0.70)	<0.001	4.81 (0.79)	4.34 (0.79)	<0.001	4.82 (0.79)	4.34 (0.79)	<0.001
Sex, n,%									
female	12,159 (42.6%)	20,916 (42.9%)	0.353	10,945 (42.5%)	10,834 (42.0%)	0.322	10,637 (42.4%)	10,418 (41.6%)	0.047
Age (years), mean, SD	68.94 (15.03)	69.45 (15.18)	<0.001	68.96 (15.07)	68.97 (14.87)	0.997	68.82 (15.04)	68.68 (14.95)	0.313
BMI, mean, SD	23.15 (4.13)	23.06 (4.19)	0.007	23.13 (4.13)	23.18 (4.20)	0.231	23.18 (4.09)	23.22 (4.19)	0.296
CCI, n,%									
0	14,435 (50.5%)	23,352 (47.9%)	<0.001	12,874 (50.0%)	12,900 (50.1%)	0.957	12,690 (50.6%)	12,782 (51.0%)	0.714
1	2,126 (7.4%)	3,906 (8.0%)		1,954 (7.6%)	1,931 (7.5%)		1,902 (7.6%)	1,854 (7.4%)	
2	8,139 (28.5%)	14,471 (29.7%)		7,410 (28.8%)	7,435 (28.9%)		7,181 (28.7%)	7,189 (28.7%)	
3 or over	3,856 (13.5%)	7,001 (14.4%)		3,532 (13.7%)	3,504 (13.6%)		3,288 (13.1%)	3,236 (12.9%)	
Smoking status (Brinkman Index), n,%									
0	14,610 (51.2%)	25,205 (51.7%)	0.020	13,189 (51.2%)	13,074 (50.7%)	0.625	12,825 (51.2%)	12,624 (50.4%)	0.284
1-399	3,167 (11.1%)	5,064 (10.4%)		2,836 (11.0%)	2,882 (11.2%)		2,768 (11.0%)	2,840 (11.3%)	
400-599	1,756 (6.1%)	3,061 (6.3%)		1,600 (6.2%)	1,570 (6.1%)		1,543 (6.2%)	1,531 (6.1%)	
600-	9,023 (31.6%)	15,400 (31.6%)		8,145 (31.6%)	8,244 (32.0%)		7,925 (31.6%)	8,066 (32.2%)	
Prehospital residence, n,%									
Home	27,374 (95.9%)	46,103 (94.6%)	<0.001	24,651 (95.7%)	24,751 (96.0%)	0.065	24,026 (95.9%)	24,119 (96.2%)	0.055
Hospital or clinic	633 (2.2%)	1,456 (3.0%)		605 (2.3%)	534 (2.1%)		559 (2.2%)	485 (1.9%)	
Long-term care	549 (1.9%)	1,171 (2.4%)		514 (2.0%)	485 (1.9%)		476 (1.9%)	457 (1.8%)	
Unconsciousness at admission	2,415 (8.5%)	5,306 (10.9%)	<0.001	2,294 (8.9%)	2,140 (8.3%)	0.016	2,054 (8.2%)	1,923 (7.7%)	0.030
Weekend admission	3,239 (11.3%)	6,081 (12.5%)	<0.001	2,969 (11.5%)	2,852 (11.1%)	0.103	2,837 (11.3%)	2,736 (10.9%)	0.151
Surgery	14,803 (51.8%)	26,334 (54.0%)	<0.001	13,392 (52.0%)	13,136 (51.0%)	0.024	13,208 (52.7%)	12,955 (51.7%)	0.024
Ambulance use at admission	4,199 (14.7%)	9,371 (19.2%)	<0.001	3,979 (15.4%)	3,632 (14.1%)	<0.001	3,686 (14.7%)	3,308 (13.2%)	<0.001
ICU stay during hospitalization	579 (2.0%)	1,517 (3.1%)	<0.001	560 (2.2%)	440 (1.7%)	<0.001	536 (2.1%)	389 (1.6%)	<0.001
Major diagnostic categories (MDCs) , n,%									
Diseases and Disorders of the Nervous System	2,122 (7.4%)	4,015 (8.2%)	<0.001	2,007 (7.8%)	1,964 (7.6%)	<0.001	1,965 (7.8%)	1,916 (7.6%)	<0.001
Diseases and Disorders of the Eye	2,410 (8.4%)	2,615 (5.4%)		1,970 (7.6%)	2,211 (8.6%)		1,936 (7.7%)	2,177 (8.7%)	
Diseases and Disorders of the Ear, Nose, Mouth and Throat	1,100 (3.9%)	1,930 (4.0%)		1,014 (3.9%)	963 (3.7%)		1,012 (4.0%)	927 (3.7%)	
Diseases and Disorders of the Respiratory System	2,374 (8.3%)	4,540 (9.3%)		2,165 (8.4%)	2,105 (8.2%)		2,018 (8.1%)	1,958 (7.8%)	
Diseases and Disorders of the Circulatory System	5,114 (17.9%)	8,128 (16.7%)		4,582 (17.8%)	4,859 (18.9%)		4,494 (17.9%)	4,760 (19.0%)	
Diseases and Disorders of the Digestive System, Hepatobiliary System, and	6,335 (22.2%)	10,353 (21.2%)		5,696 (22.1%)	5,714 (22.2%)		5,466 (21.8%)	5,575 (22.2%)	
Diseases and Disorders of the Musculoskeletal System and Connective Tissues	1,610 (5.6%)	3,548 (7.3%)		1,503 (5.8%)	1,268 (4.9%)		1,489 (5.9%)	1,245 (5.0%)	
Diseases and Disorders of the Skin and Subcutaneous Tissue	591 (2.1%)	955 (2.0%)		519 (2.0%)	516 (2.0%)		524 (2.1%)	541 (2.2%)	
Diseases and Disorders of the Breast	296 (1.0%)	520 (1.1%)		275 (1.1%)	271 (1.1%)		275 (1.1%)	263 (1.0%)	
Diseases and Disorders of the Endocrine, Nutritional and Metabolic System	876 (3.1%)	1,731 (3.6%)		829 (3.2%)	737 (2.9%)		829 (3.3%)	714 (2.8%)	
Diseases and Disorders of the Kidney, Urinary Tract and Male Reproductive System	2,721 (9.5%)	4,719 (9.7%)		2,478 (9.6%)	2,589 (10.0%)		2,429 (9.7%)	2,568 (10.2%)	
Diseases and Disorders Pertaining to the Female Reproductive System, Pregnancy, Childbirth, and Puerperium	828 (2.9%)	1,198 (2.5%)		713 (2.8%)	698 (2.7%)		679 (2.7%)	691 (2.8%)	
Diseases and Disorders of the Blood, Blood Forming Organ and Myeloproliferative Diseases and Disorders	637 (2.2%)	1,081 (2.2%)		586 (2.3%)	582 (2.3%)		526 (2.1%)	497 (2.0%)	
Pediatric Diseases and Disorder	55 (0.2%)	87 (0.2%)		46 (0.2%)	48 (0.2%)		50 (0.2%)	53 (0.2%)	
Trauma, Burns, and Poisonings	1,018 (3.6%)	2,386 (4.9%)		962 (3.7%)	821 (3.2%)		954 (3.8%)	775 (3.1%)	
Mental Diseases and Disorders	38 (0.1%)	52 (0.1%)		34 (0.1%)	38 (0.1%)		37 (0.1%)	41 (0.2%)	
Other Diseases and Disorders	431 (1.5%)	872 (1.8%)		391 (1.5%)	386 (1.5%)		378 (1.5%)	360 (1.4%)	
Severity of a Patient's Condition and Extent of a Patient's Need for Medical/Nursing Care index[c]									
Item A_Monitoring and treatment									
0	21,887 (76.6%)	36,755 (75.4%)	<0.001	19,687 (76.4%)	19,723 (76.5%)	0.109	19,241 (76.8%)	19,249 (76.8%)	0.081
1	2,696 (9.4%)	4,446 (9.1%)		2,436 (9.5%)	2,549 (9.9%)		2,402 (9.6%)	2,532 (10.1%)	
2	2,099 (7.4%)	3,922 (8.0%)		1,928 (7.5%)	1,874 (7.3%)		1,844 (7.4%)	1,797 (7.2%)	
3 or over	1,874 (6.6%)	3,607 (7.4%)		1,719 (6.7%)	1,624 (6.3%)		1,574 (6.3%)	1,483 (5.9%)	
Item B_Patients' functional status									
0	17,878 (62.6%)	29,890 (61.3%)	<0.001	16,062 (62.3%)	16,254 (63.1%)	0.087	15,792 (63.0%)	15,986 (63.8%)	0.069
1	3,092 (10.8%)	5,074 (10.4%)		2,786 (10.8%)	2,819 (10.9%)		2,728 (10.9%)	2,720 (10.9%)	
2	1,807 (6.3%)	2,797 (5.7%)		1,589 (6.2%)	1,597 (6.2%)		1,541 (6.1%)	1,583 (6.3%)	
3 or over	5,779 (20.2%)	10,969 (22.5%)		5,333 (20.7%)	5,100 (19.8%)		5,000 (20.0%)	4,772 (19.0%)	
Item C_Medical management, such as medical treatment related to surgery and emergency care									
0	27,880 (97.6%)	47,646 (97.8%)	0.199	25,150 (97.6%)	25,160 (97.6%)	0.773	24,452 (97.6%)	24,452 (97.6%)	1.000

© 2026 Morioka N et al. *JAMA Network Open*.

1 or over	676 (2.4%)	1,084 (2.2%)		620 (2.4%)	610 (2.4%)		609 (2.4%)	609 (2.4%)	
**Ward level variables**									
Number of inpatients on a ward during hospitalization, mean, SD	36.30 (6.50)	39.23 (6.08)	<0.001	37.12 (6.08)	36.85 (6.73)	<0.001	37.11 (6.06)	36.85 (6.71)	<0.001
Median age of inpatients on a ward during hospitalization (years), mean, SD	71.11 (3.43)	71.33 (3.49)	<0.001	71.17 (3.38)	71.23 (3.58)	0.083	71.15 (3.38)	71.22 (3.57)	0.045
a ward during hospitalization (%), mean, SD	21.87 (10.66)	20.82 (9.43)	<0.001	21.40 (10.24)	21.63 (9.80)	0.010	21.38 (10.27)	21.65 (9.81)	0.003

[a] In the propensity score estimation, adjustments were made for age, age squared, Charlson Comorbidity Index (CCI), smoking index, body mass index (BMI), place of residence before admission, ICU stay, unconsciousness at admission, ambulance use, weekend admission, place after discharge (for the readmission model only), main diagnosis category, the Japanese Severity and Medical/Nursing Care Needs Score (Items A, B, and C) at admission, percentage of severe inpatients in the ward, median age of inpatients in the ward, number of inpatients in the ward, and hospital fixed effects (dummy variables).

[b] Chi-square test or t-test

[c] The Severity of a Patient's Condition and Extent of a Patient's Need for Medical/Nursing Care for General Wards comprises 21 items. Item A (7 items) assesses the need for specialized nursing care, including monitoring and treatment; Item B (7 items) evaluates patients' functional status, such as activities of daily living (ADLs), that influence health service use; and Item C (7 items) measures the need for medical management, including treatments related to surgery and emergency care.

© 2026 Morioka N et al. *JAMA Network Open*.

eTable 8. Characteristics of Individuals Before and After Propensity Score Matching for 24-Hour Period Understaffing Using the Annual Median Minus 5% as the Cutoff

	Before PS matching			After PS[a] matching for in-hospital death and LOS			After PS[a] matching for readmission		
	Adequately staffed group (>=annual median -5% point) (N=59,432)	Understaffed group (belowannual median -5% point) (N=17,854)	P-value[b]	Adequately staffed group (>=annual median -5% point) (N=17,654)	Understaffed group (belowannual median -5% point) (N=17,654)	P-value[b]	Adequately staffed group (>=annual median -5% point) (N=17,087)	Understaffed group (belowannual median -5% point) (N=17,087)	P-value[b]
NHPPD	4.58 (0.84)	3.86 (0.61)	<0.001	4.38 (0.71)	3.87 (0.62)	<0.001	4.39 (0.71)	3.87 (0.62)	<0.001
Sex, n,%									
female	25,408 (42.8%)	7,667 (42.9%)	0.650	7,657 (43.4%)	7,551 (42.8%)	0.255	7,377 (43.2%)	7,333 (42.9%)	0.631
Age (years), mean, SD	69.30 (15.02)	69.14 (15.48)	0.228	69.10 (15.66)	69.21 (15.41)	0.498	68.94 (15.59)	68.94 (15.46)	0.984
BMI, mean, SD	23.09 (4.17)	23.12 (4.18)	0.286	23.08 (4.15)	23.12 (4.17)	0.351	23.19 (4.21)	23.19 (4.15)	0.909
CCI, n,%									
0	29,132 (49.0%)	8,655 (48.5%)	0.165	8,742 (49.5%)	8,530 (48.3%)	0.151	8,579 (50.2%)	8,418 (49.3%)	0.108
1	4,652 (7.8%)	1,380 (7.7%)		1,342 (7.6%)	1,359 (7.7%)		1,389 (8.1%)	1,331 (7.8%)	
2	17,267 (29.1%)	5,343 (29.9%)		5,149 (29.2%)	5,301 (30.0%)		4,953 (29.0%)	5,107 (29.9%)	
3 or over	8,381 (14.1%)	2,476 (13.9%)		2,421 (13.7%)	2,464 (14.0%)		2,166 (12.7%)	2,231 (13.1%)	
Smoking status (Brinkman Index), n,%									
0	30,471 (51.3%)	9,344 (52.3%)	0.008	9,305 (52.7%)	9,216 (52.2%)	0.818	8,967 (52.5%)	8,937 (52.3%)	0.913
1-399	6,338 (10.7%)	1,893 (10.6%)		1,849 (10.5%)	1,877 (10.6%)		1,790 (10.5%)	1,828 (10.7%)	
400-599	3,666 (6.2%)	1,151 (6.4%)		1,123 (6.4%)	1,137 (6.4%)		1,096 (6.4%)	1,106 (6.5%)	
600-	18,957 (31.9%)	5,466 (30.6%)		5,377 (30.5%)	5,424 (30.7%)		5,234 (30.6%)	5,216 (30.5%)	
Prehospital residence, n,%									
Home	56,555 (95.2%)	16,922 (94.8%)	0.013	16,760 (94.9%)	16,738 (94.8%)	0.615	16,275 (95.2%)	16,263 (95.2%)	0.346
Hospital or clinic	1,605 (2.7%)	484 (2.7%)		452 (2.6%)	481 (2.7%)		404 (2.4%)	439 (2.6%)	
Long-term care	1,272 (2.1%)	448 (2.5%)		442 (2.5%)	435 (2.5%)		408 (2.4%)	385 (2.3%)	
Unconsciousness at admission	5,741 (9.7%)	1,980 (11.1%)	<0.001	2,025 (11.5%)	1,932 (10.9%)	0.117	1,759 (10.3%)	1,708 (10.0%)	0.361
Weekend admission	6,959 (11.7%)	2,361 (13.2%)	<0.001	2,394 (13.6%)	2,319 (13.1%)	0.241	2,215 (13.0%)	2,190 (12.8%)	0.687
Surgery	31,622 (53.2%)	9,515 (53.3%)	0.839	9,359 (53.0%)	9,413 (53.3%)	0.565	9,231 (54.0%)	9,288 (54.4%)	0.536
Ambulance use at admission	9,996 (16.8%)	3,574 (20.0%)	<0.001	3,561 (20.2%)	3,505 (19.9%)	0.456	3,284 (19.2%)	3,217 (18.8%)	0.356
ICU stay during hospitalization	1,643 (2.8%)	453 (2.5%)	0.101	437 (2.5%)	453 (2.6%)	0.587	383 (2.2%)	424 (2.5%)	0.144
Major diagnostic categories (MDCs) , n,%									
Diseases and Disorders of the Nervous System	4,616 (7.8%)	1,521 (8.5%)	<0.001	1,524 (8.6%)	1,485 (8.4%)	0.240	1,392 (8.1%)	1,428 (8.4%)	0.163
Diseases and Disorders of the Eye	4,008 (6.7%)	1,017 (5.7%)		1,041 (5.9%)	1,005 (5.7%)		1,015 (5.9%)	1,005 (5.9%)	
Diseases and Disorders of the Ear, Nose, Mouth and Throat	2,358 (4.0%)	672 (3.8%)		695 (3.9%)	671 (3.8%)		731 (4.3%)	669 (3.9%)	
Diseases and Disorders of the Respiratory System	5,349 (9.0%)	1,565 (8.8%)		1,532 (8.7%)	1,559 (8.8%)		1,429 (8.4%)	1,431 (8.4%)	
Diseases and Disorders of the Circulatory System	10,087 (17.0%)	3,155 (17.7%)		2,904 (16.4%)	3,143 (17.8%)		2,903 (17.0%)	3,065 (17.9%)	
System, Hepatobiliary System, and Pancreas	12,712 (21.4%)	3,976 (22.3%)		3,937 (22.3%)	3,961 (22.4%)		3,655 (21.4%)	3,797 (22.2%)	
Diseases and Disorders of the Musculoskeletal System and Connective Tissues	4,169 (7.0%)	989 (5.5%)		973 (5.5%)	989 (5.6%)		992 (5.8%)	976 (5.7%)	
Diseases and Disorders of the Skin and Subcutaneous Tissue	1,131 (1.9%)	415 (2.3%)		430 (2.4%)	393 (2.2%)		416 (2.4%)	391 (2.3%)	
Diseases and Disorders of the Breast	613 (1.0%)	203 (1.1%)		196 (1.1%)	202 (1.1%)		202 (1.2%)	197 (1.2%)	
Diseases and Disorders of the Endocrine, Nutritional and Metabolic System	1,975 (3.3%)	632 (3.5%)		663 (3.8%)	613 (3.5%)		694 (4.1%)	613 (3.6%)	
Diseases and Disorders of the Kidney, Urinary Tract and Male Reproductive System	5,781 (9.7%)	1,659 (9.3%)		1,686 (9.6%)	1,643 (9.3%)		1,629 (9.5%)	1,608 (9.4%)	
Diseases and Disorders Pertaining to the Female Reproductive System, Pregnancy, Childbirth, and Puerperium	1,466 (2.5%)	560 (3.1%)		575 (3.3%)	513 (2.9%)		552 (3.2%)	502 (2.9%)	
Diseases and Disorders of the Blood, Blood Forming Organ and Myeloproliferative Diseases and Disorders	1,373 (2.3%)	345 (1.9%)		350 (2.0%)	345 (2.0%)		335 (2.0%)	311 (1.8%)	
Pediatric Diseases and Disorder	113 (0.2%)	29 (0.2%)		28 (0.2%)	29 (0.2%)		24 (0.1%)	28 (0.2%)	
Trauma, Burns, and Poisonings	2,606 (4.4%)	798 (4.5%)		799 (4.5%)	788 (4.5%)		816 (4.8%)	773 (4.5%)	
Mental Diseases and Disorders	66 (0.1%)	24 (0.1%)		22 (0.1%)	24 (0.1%)		23 (0.1%)	24 (0.1%)	
Other Diseases and Disorders	1,009 (1.7%)	294 (1.6%)		299 (1.7%)	291 (1.6%)		279 (1.6%)	269 (1.6%)	
Severity of a Patient's Condition and Extent of a Patient's Need for Medical/Nursing Care index[c]									
Item A_Monitoring and treatment									
0	45,259 (76.2%)	13,383 (75.0%)	<0.001	13,209 (74.8%)	13,253 (75.1%)	0.494	12,832 (75.1%)	12,946 (75.8%)	0.551
1	5,493 (9.2%)	1,649 (9.2%)		1,590 (9.0%)	1,632 (9.2%)		1,639 (9.6%)	1,587 (9.3%)	
2	4,587 (7.7%)	1,434 (8.0%)		1,431 (8.1%)	1,413 (8.0%)		1,369 (8.0%)	1,332 (7.8%)	
3 or over	4,093 (6.9%)	1,388 (7.8%)		1,424 (8.1%)	1,356 (7.7%)		1,247 (7.3%)	1,222 (7.2%)	
Item B_Patients' functional status									
0	36,646 (61.7%)	11,122 (62.3%)	<0.001	10,829 (61.3%)	10,995 (62.3%)	0.322	10,641 (62.3%)	10,770 (63.0%)	0.412
1	6,433 (10.8%)	1,733 (9.7%)		1,776 (10.1%)	1,727 (9.8%)		1,685 (9.9%)	1,695 (9.9%)	
2	3,588 (6.0%)	1,016 (5.7%)		1,017 (5.8%)	1,010 (5.7%)		1,005 (5.9%)	973 (5.7%)	
3 or over	12,765 (21.5%)	3,983 (22.3%)		4,032 (22.8%)	3,922 (22.2%)		3,756 (22.0%)	3,649 (21.4%)	

© 2026 Morioka N et al. *JAMA Network Open*.

Item C_Medical management, such as medical treatment related to surgery and emergency care									
0	58,125 (97.8%)	17,401 (97.5%)	0.008	17,189 (97.4%)	17,215 (97.5%)	0.381	16,637 (97.4%)	16,650 (97.4%)	0.658
1 or over	1,307 (2.2%)	453 (2.5%)		465 (2.6%)	439 (2.5%)		450 (2.6%)	437 (2.6%)	
**Ward level variables**									
Number of inpatients on a ward during hospitalization, mean, SD	37.68 (6.45)	39.71 (5.96)	<0.001	39.40 (5.53)	39.63 (5.93)	<0.001	39.41 (5.46)	39.61 (5.93)	0.001
Median age of inpatients on a ward during hospitalization (years), mean, SD	71.19 (3.44)	71.45 (3.55)	<0.001	71.42 (3.40)	71.44 (3.55)	0.597	71.40 (3.38)	71.41 (3.55)	0.623
a ward during hospitalization (%), mean, SD	21.43 (10.01)	20.46 (9.58)	<0.001	19.81 (9.53)	20.52 (9.57)	<0.001	19.91 (9.57)	20.50 (9.59)	<0.001

[a] In the propensity score estimation, adjustments were made for age, age squared, Charlson Comorbidity Index (CCI), smoking index, body mass index (BMI), place of residence before admission, ICU stay, unconsciousness at admission, ambulance use, weekend admission, place after discharge (for the readmission model only), main diagnosis category, the Japanese Severity and Medical/Nursing Care Needs Score (Items A, B, and C) at admission, percentage of severe inpatients in the ward, median age of inpatients in the ward, number of inpatients in the ward, and hospital fixed effects (dummy variables).

[b] Chi-square test or t-test

[c] The Severity of a Patient's Condition and Extent of a Patient's Need for Medical/Nursing Care for General Wards comprises 21 items. Item A (7 items) assesses the need for specialized nursing care, including monitoring and treatment; Item B (7 items) evaluates patients' functional status, such as activities of daily living (ADLs), that influence health service use; and Item C (7 items) measures the need for medical management, including treatments related to surgery and emergency care.

© 2026 Morioka N et al. *JAMA Network Open*.

eTable 9. Characteristics of Individuals Before and After Propensity Score Matching for 24-Hour Period Understaffing Using the Annual Median Minus 10% as the Cutoff

	Before PS matching			After PS[a] matching for in-hospital death and LOS			After PS[a] matching for readmission		
	Adequately staffed group (>=annual median -10% point) (N=69,751)	Understaffed group (belowannual median -10% point) (N=7,535)	P-value[b]	Adequately staffed group (>=annual median -10% point) (N=7,535)	Understaffed group (belowannual median -10% point) (N=7,535)	P-value[b]	Adequately staffed group (>=annual median -10% point) (N=7,305)	Understaffed group (belowannual median -10% point) (N=7,305)	P-value[b]
NHPPD	4.49 (0.84)	3.73 (0.60)	<0.001	4.34 (0.71)	3.73 (0.60)	<0.001	4.34 (0.73)	3.73 (0.60)	<0.001
Sex, n,%									
female	29,868 (42.8%)	3,207 (42.6%)	0.665	3,216 (42.7%)	3,207 (42.6%)	0.882	3,161 (43.3%)	3,115 (42.6%)	0.442
Age (years), mean, SD	69.37 (15.04)	68.27 (15.89)	<0.001	68.20 (16.01)	68.27 (15.89)	0.795	67.81 (16.03)	67.96 (15.91)	0.563
BMI, mean, SD	23.08 (4.17)	23.24 (4.15)	0.001	23.16 (4.21)	23.24 (4.15)	0.198	23.28 (4.26)	23.31 (4.12)	0.635
CCI, n,%									
0	34,007 (48.8%)	3,780 (50.2%)	0.028	3,723 (49.4%)	3,780 (50.2%)	0.370	3,717 (50.9%)	3,735 (51.1%)	0.623
1	5,458 (7.8%)	574 (7.6%)		614 (8.1%)	574 (7.6%)		591 (8.1%)	558 (7.6%)	
2	20,412 (29.3%)	2,198 (29.2%)		2,167 (28.8%)	2,198 (29.2%)		2,077 (28.4%)	2,118 (29.0%)	
3 or over	9,874 (14.2%)	983 (13.0%)		1,031 (13.7%)	983 (13.0%)		920 (12.6%)	894 (12.2%)	
Smoking status (Brinkman Index), n,%									
0	35,831 (51.4%)	3,984 (52.9%)	0.003	3,924 (52.1%)	3,984 (52.9%)	0.730	3,899 (53.4%)	3,866 (52.9%)	0.918
1-399	7,416 (10.6%)	815 (10.8%)		849 (11.3%)	815 (10.8%)		796 (10.9%)	796 (10.9%)	
400-599	4,322 (6.2%)	495 (6.6%)		504 (6.7%)	495 (6.6%)		489 (6.7%)	484 (6.6%)	
600-	22,182 (31.8%)	2,241 (29.7%)		2,258 (30.0%)	2,241 (29.7%)		2,121 (29.0%)	2,159 (29.6%)	
Prehospital residence, n,%									
Home	66,268 (95.0%)	7,209 (95.7%)	0.003	7,214 (95.7%)	7,209 (95.7%)	0.972	7,011 (96.0%)	7,020 (96.1%)	0.871
Hospital or clinic	1,931 (2.8%)	158 (2.1%)		154 (2.0%)	158 (2.1%)		154 (2.1%)	145 (2.0%)	
Long-term care	1,552 (2.2%)	168 (2.2%)		167 (2.2%)	168 (2.2%)		140 (1.9%)	140 (1.9%)	
Unconsciousness at admission	6,909 (9.9%)	812 (10.8%)	0.017	842 (11.2%)	812 (10.8%)	0.434	728 (10.0%)	708 (9.7%)	0.578
Weekend admission	8,280 (11.9%)	1,040 (13.8%)	<0.001	1,018 (13.5%)	1,040 (13.8%)	0.602	1,018 (13.9%)	985 (13.5%)	0.427
Surgery	37,148 (53.3%)	3,989 (52.9%)	0.599	3,944 (52.3%)	3,989 (52.9%)	0.463	3,844 (52.6%)	3,944 (54.0%)	0.097
Ambulance use at admission	12,055 (17.3%)	1,515 (20.1%)	<0.001	1,490 (19.8%)	1,515 (20.1%)	0.610	1,405 (19.2%)	1,386 (19.0%)	0.689
ICU stay during hospitalization	1,944 (2.8%)	152 (2.0%)	<0.001	164 (2.2%)	152 (2.0%)	0.495	135 (1.8%)	142 (1.9%)	0.671
Major diagnostic categories (MDCs) , n,%									
Diseases and Disorders of the Nervous System	5,482 (7.9%)	655 (8.7%)	<0.001	620 (8.2%)	655 (8.7%)	0.453	594 (8.1%)	629 (8.6%)	0.729
Diseases and Disorders of the Eye	4,486 (6.4%)	539 (7.2%)		544 (7.2%)	539 (7.2%)		560 (7.7%)	539 (7.4%)	
Diseases and Disorders of the Ear, Nose, Mouth and Throat	2,737 (3.9%)	293 (3.9%)		302 (4.0%)	293 (3.9%)		284 (3.9%)	293 (4.0%)	
Diseases and Disorders of the Respiratory System	6,373 (9.1%)	541 (7.2%)		578 (7.7%)	541 (7.2%)		486 (6.7%)	488 (6.7%)	
Diseases and Disorders of the Circulatory System	11,844 (17.0%)	1,398 (18.6%)		1,307 (17.3%)	1,398 (18.6%)		1,275 (17.5%)	1,363 (18.7%)	
System, Hepatobiliary System, and Pancreas	15,002 (21.5%)	1,686 (22.4%)		1,679 (22.3%)	1,686 (22.4%)		1,657 (22.7%)	1,625 (22.2%)	
Diseases and Disorders of the Musculoskeletal System and Connective Tissues	4,867 (7.0%)	291 (3.9%)		314 (4.2%)	291 (3.9%)		334 (4.6%)	291 (4.0%)	
Diseases and Disorders of the Skin and Subcutaneous Tissue	1,359 (1.9%)	187 (2.5%)		198 (2.6%)	187 (2.5%)		186 (2.5%)	186 (2.5%)	
Diseases and Disorders of the Breast	729 (1.0%)	87 (1.2%)		80 (1.1%)	87 (1.2%)		84 (1.1%)	84 (1.1%)	
Diseases and Disorders of the Endocrine, Nutritional and Metabolic System	2,323 (3.3%)	284 (3.8%)		293 (3.9%)	284 (3.8%)		287 (3.9%)	283 (3.9%)	
Diseases and Disorders of the Kidney, Urinary Tract and Male Reproductive System	6,728 (9.6%)	712 (9.4%)		756 (10.0%)	712 (9.4%)		685 (9.4%)	695 (9.5%)	
Diseases and Disorders Pertaining to the Female Reproductive System, Pregnancy, Childbirth, and Puerperium	1,759 (2.5%)	267 (3.5%)		302 (4.0%)	267 (3.5%)		294 (4.0%)	262 (3.6%)	
Diseases and Disorders of the Blood, Blood Forming Organ and Myeloproliferative Diseases and Disorders	1,573 (2.3%)	145 (1.9%)		163 (2.2%)	145 (1.9%)		134 (1.8%)	130 (1.8%)	
Pediatric Diseases and Disorder	131 (0.2%)	11 (0.1%)		8 (0.1%)	11 (0.1%)		6 (0.1%)	11 (0.2%)	
Trauma, Burns, and Poisonings	3,091 (4.4%)	313 (4.2%)		268 (3.6%)	313 (4.2%)		307 (4.2%)	306 (4.2%)	
Mental Diseases and Disorders	71 (0.1%)	19 (0.3%)		15 (0.2%)	19 (0.3%)		25 (0.3%)	19 (0.3%)	
Other Diseases and Disorders	1,196 (1.7%)	107 (1.4%)		108 (1.4%)	107 (1.4%)		107 (1.5%)	101 (1.4%)	
Severity of a Patient's Condition and Extent of a Patient's Need for Medical/Nursing Care index[c]									
Item A_Monitoring and treatment									
0	53,010 (76.0%)	5,632 (74.7%)	0.101	5,637 (74.8%)	5,632 (74.7%)	0.812	5,491 (75.2%)	5,497 (75.2%)	0.891
1	6,406 (9.2%)	736 (9.8%)		705 (9.4%)	736 (9.8%)		708 (9.7%)	727 (10.0%)	
2	5,418 (7.8%)	603 (8.0%)		617 (8.2%)	603 (8.0%)		594 (8.1%)	574 (7.9%)	
3 or over	4,917 (7.0%)	564 (7.5%)		576 (7.6%)	564 (7.5%)		512 (7.0%)	507 (6.9%)	
Item B_Patients' functional status									
0	42,983 (61.6%)	4,785 (63.5%)	0.003	4,760 (63.2%)	4,785 (63.5%)	0.911	4,604 (63.0%)	4,673 (64.0%)	0.676
1	7,444 (10.7%)	722 (9.6%)		736 (9.8%)	722 (9.6%)		735 (10.1%)	708 (9.7%)	
2	4,183 (6.0%)	421 (5.6%)		409 (5.4%)	421 (5.6%)		416 (5.7%)	413 (5.7%)	
3 or over	15,141 (21.7%)	1,607 (21.3%)		1,630 (21.6%)	1,607 (21.3%)		1,550 (21.2%)	1,511 (20.7%)	

© 2026 Morioka N et al. *JAMA Network Open*.

Item C_Medical management, such as medical treatment related to surgery and emergency care									
0	68,190 (97.8%)	7,336 (97.4%)	0.026	7,346 (97.5%)	7,336 (97.4%)	0.607	7,088 (97.0%)	7,106 (97.3%)	0.371
1 or over	1,561 (2.2%)	199 (2.6%)		189 (2.5%)	199 (2.6%)		217 (3.0%)	199 (2.7%)	
**Ward level variables**									
Number of inpatients on a ward during hospitalization, mean, SD	37.98 (6.40)	39.63 (6.17)	<0.001	39.38 (5.43)	39.63 (6.17)	0.011	39.40 (5.48)	39.62 (6.17)	0.020
Median age of inpatients on a ward during hospitalization (years), mean, SD	71.24 (3.44)	71.37 (3.69)	0.002	71.33 (3.43)	71.37 (3.69)	0.503	71.19 (3.38)	71.33 (3.69)	0.013
a ward during hospitalization (%), mean, SD	21.34 (9.91)	20.05 (9.93)	<0.001	19.43 (9.69)	20.05 (9.93)	<0.001	19.53 (9.91)	20.04 (9.94)	0.002

[a] In the propensity score estimation, adjustments were made for age, age squared, Charlson Comorbidity Index (CCI), smoking index, body mass index (BMI), place of residence before admission, ICU stay, unconsciousness at admission, ambulance use, weekend admission, place after discharge (for the readmission model only), main diagnosis category, the Japanese Severity and Medical/Nursing Care Needs Score (Items A, B, and C) at admission, percentage of severe inpatients in the ward, median age of inpatients in the ward, number of inpatients in the ward, and hospital fixed effects (dummy variables).

[b] Chi-square test or t-test

[c] The Severity of a Patient's Condition and Extent of a Patient's Need for Medical/Nursing Care for General Wards comprises 21 items. Item A (7 items) assesses the need for specialized nursing care, including monitoring and treatment; Item B (7 items) evaluates patients' functional status, such as activities of daily living (ADLs), that influence health service use; and Item C (7 items) measures the need for medical management, including treatments related to surgery and emergency care.

© 2026 Morioka N et al. *JAMA Network Open*.

eTable 10. Characteristics of Individuals Before and After Propensity Score Matching for 24-Hour Period Understaffing Using the Annual Median Minus 15% as the Cutoff

	Before PS matching			After PS[a] matching for in-hospital death and LOS			After PS[a] matching for readmission		
	Adequately staffed group (>=annual median -15% point) (N=74,478)	Understaffed group (belowannual median -15% point) (N=2,808)	P-value[b]	Adequately staffed group (>=annual median -15% point) (N=2,808)	Understaffed group (belowannual median -15% point) (N=2,808)	P-value[b]	Adequately staffed group (>=annual median -15% point) (N=2,712)	Understaffed group (belowannual median -15% point) (N=2,712)	P-value[b]
NHPPD	4.4 (0.8)	3.6 (0.6)	<0.001	4.29 (0.70)	3.55 (0.60)	<0.001	4.32 (0.71)	3.56 (0.60)	<0.001
Sex, n,%									
female	31,895 (42.8%)	1,180 (42.0%)	0.399	1,175 (41.8%)	1,180 (42.0%)	0.892	1,136 (41.9%)	1,141 (42.1%)	0.891
Age (years), mean, SD	69.3 (15.1)	67.4 (16.5)	<0.001	67.63 (16.70)	67.43 (16.53)	0.660	66.93 (16.78)	67.02 (16.56)	0.829
BMI, mean, SD	23.1 (4.2)	23.2 (4.1)	0.069	23.22 (4.35)	23.24 (4.09)	0.916	23.18 (4.13)	23.31 (4.03)	0.251
CCI, n,%									
0	36,285 (48.7%)	1,502 (53.5%)	<0.001	1,527 (54.4%)	1,502 (53.5%)	0.777	1,453 (53.6%)	1,483 (54.7%)	0.775
1	5,836 (7.8%)	196 (7.0%)		205 (7.3%)	196 (7.0%)		184 (6.8%)	191 (7.0%)	
2	21,840 (29.3%)	770 (27.4%)		755 (26.9%)	770 (27.4%)		756 (27.9%)	733 (27.0%)	
3 or over	10,517 (14.1%)	340 (12.1%)		321 (11.4%)	340 (12.1%)		319 (11.8%)	305 (11.2%)	
Smoking status (Brinkman Index), n,%									
0	38,311 (51.4%)	1,504 (53.6%)	0.027	1,527 (54.4%)	1,504 (53.6%)	0.753	1,424 (52.5%)	1,451 (53.5%)	0.906
1-399	7,939 (10.7%)	292 (10.4%)		270 (9.6%)	292 (10.4%)		291 (10.7%)	284 (10.5%)	
400-599	4,626 (6.2%)	191 (6.8%)		198 (7.1%)	191 (6.8%)		193 (7.1%)	187 (6.9%)	
600-	23,602 (31.7%)	821 (29.2%)		813 (29.0%)	821 (29.2%)		804 (29.6%)	790 (29.1%)	
Prehospital residence, n,%									
Home	70,777 (95.0%)	2,700 (96.2%)	0.009	2,703 (96.3%)	2,700 (96.2%)	0.955	2,613 (96.3%)	2,619 (96.6%)	0.907
Hospital or clinic	2,038 (2.7%)	51 (1.8%)		48 (1.7%)	51 (1.8%)		51 (1.9%)	48 (1.8%)	
Long-term care	1,663 (2.2%)	57 (2.0%)		57 (2.0%)	57 (2.0%)		48 (1.8%)	45 (1.7%)	
Unconsciousness at admission	7,412 (10.0%)	309 (11.0%)	0.068	275 (9.8%)	309 (11.0%)	0.137	247 (9.1%)	257 (9.5%)	0.640
Weekend admission	8,880 (11.9%)	440 (15.7%)	<0.001	446 (15.9%)	440 (15.7%)	0.826	406 (15.0%)	413 (15.2%)	0.791
Surgery	39,686 (53.3%)	1,451 (51.7%)	0.093	1,463 (52.1%)	1,451 (51.7%)	0.749	1,424 (52.5%)	1,431 (52.8%)	0.849
Ambulance use at admission	12,979 (17.4%)	591 (21.0%)	<0.001	586 (20.9%)	591 (21.0%)	0.870	497 (18.3%)	529 (19.5%)	0.267
ICU stay during hospitalization	2,053 (2.8%)	43 (1.5%)	<0.001	37 (1.3%)	43 (1.5%)	0.499	34 (1.3%)	40 (1.5%)	0.482
Major diagnostic categories (MDCs) , n,%									
Diseases and Disorders of the Nervous System	5,885 (7.9%)	252 (9.0%)	<0.001	218 (7.8%)	252 (9.0%)	0.477	227 (8.4%)	240 (8.8%)	0.946
Diseases and Disorders of the Eye	4,818 (6.5%)	207 (7.4%)		238 (8.5%)	207 (7.4%)		198 (7.3%)	207 (7.6%)	
Diseases and Disorders of the Ear, Nose, Mouth and Throat	2,908 (3.9%)	122 (4.3%)		108 (3.8%)	122 (4.3%)		136 (5.0%)	122 (4.5%)	
Diseases and Disorders of the Respiratory System	6,743 (9.1%)	171 (6.1%)		159 (5.7%)	171 (6.1%)		155 (5.7%)	148 (5.5%)	
Diseases and Disorders of the Circulatory System	12,706 (17.1%)	536 (19.1%)		523 (18.6%)	536 (19.1%)		480 (17.7%)	526 (19.4%)	
System, Hepatobiliary System, and Pancreas	16,062 (21.6%)	626 (22.3%)		618 (22.0%)	626 (22.3%)		623 (23.0%)	602 (22.2%)	
Diseases and Disorders of the Musculoskeletal System and Connective Tissues	5,078 (6.8%)	80 (2.8%)		108 (3.8%)	80 (2.8%)		89 (3.3%)	80 (2.9%)	
Diseases and Disorders of the Skin and Subcutaneous Tissue	1,460 (2.0%)	86 (3.1%)		81 (2.9%)	86 (3.1%)		90 (3.3%)	85 (3.1%)	
Diseases and Disorders of the Breast	781 (1.0%)	35 (1.2%)		39 (1.4%)	35 (1.2%)		40 (1.5%)	33 (1.2%)	
Diseases and Disorders of the Endocrine, Nutritional and Metabolic System	2,503 (3.4%)	104 (3.7%)		101 (3.6%)	104 (3.7%)		112 (4.1%)	103 (3.8%)	
Diseases and Disorders of the Kidney, Urinary Tract and Male Reproductive System	7,173 (9.6%)	267 (9.5%)		260 (9.3%)	267 (9.5%)		252 (9.3%)	263 (9.7%)	
Diseases and Disorders Pertaining to the Female Reproductive System, Pregnancy, Childbirth, and Puerperium	1,900 (2.6%)	126 (4.5%)		132 (4.7%)	126 (4.5%)		137 (5.1%)	123 (4.5%)	
Diseases and Disorders of the Blood, Blood Forming Organ and Myeloproliferative Diseases and Disorders	1,679 (2.3%)	39 (1.4%)		51 (1.8%)	39 (1.4%)		32 (1.2%)	29 (1.1%)	
Pediatric Diseases and Disorder	139 (0.2%)	3 (0.1%)		2 (0.1%)	3 (0.1%)		1 (0.0%)	3 (0.1%)	
Trauma, Burns, and Poisonings	3,300 (4.4%)	104 (3.7%)		116 (4.1%)	104 (3.7%)		95 (3.5%)	102 (3.8%)	
Mental Diseases and Disorders	78 (0.1%)	12 (0.4%)		7 (0.2%)	12 (0.4%)		13 (0.5%)	12 (0.4%)	
Other Diseases and Disorders	1,265 (1.7%)	38 (1.4%)		47 (1.7%)	38 (1.4%)		32 (1.2%)	34 (1.3%)	
Severity of a Patient's Condition and Extent of a Patient's Need for Medical/Nursing Care index[c]									
Item A_Monitoring and treatment									
0	56,545 (75.9%)	2,097 (74.7%)	0.474	2,117 (75.4%)	2,097 (74.7%)	0.894	2,058 (75.9%)	2,038 (75.1%)	0.864
1	6,869 (9.2%)	273 (9.7%)		264 (9.4%)	273 (9.7%)		258 (9.5%)	271 (10.0%)	
2	5,787 (7.8%)	234 (8.3%)		235 (8.4%)	234 (8.3%)		213 (7.9%)	224 (8.3%)	
3 or over	5,277 (7.1%)	204 (7.3%)		192 (6.8%)	204 (7.3%)		183 (6.7%)	179 (6.6%)	
Item B_Patients' functional status									
0	45,980 (61.7%)	1,788 (63.7%)	0.103	1,782 (63.5%)	1,788 (63.7%)	0.946	1,727 (63.7%)	1,736 (64.0%)	0.813
1	7,902 (10.6%)	264 (9.4%)		263 (9.4%)	264 (9.4%)		259 (9.6%)	261 (9.6%)	
2	4,435 (6.0%)	169 (6.0%)		180 (6.4%)	169 (6.0%)		155 (5.7%)	167 (6.2%)	
3 or over	16,161 (21.7%)	587 (20.9%)		583 (20.8%)	587 (20.9%)		571 (21.1%)	548 (20.2%)	
Item C_Medical management, such as medical treatment related to surgery and emergency care									
0	72,793 (97.7%)	2,733 (97.3%)	0.154	2,739 (97.5%)	2,733 (97.3%)	0.612	2,643 (97.5%)	2,637 (97.2%)	0.612

© 2026 Morioka N et al. *JAMA Network Open*.

1 or over	1,685 (2.3%)	75 (2.7%)		69 (2.5%)	75 (2.7%)		69 (2.5%)	75 (2.8%)	
**Ward level variables**									
Average number of inpatients during the hospitalization, mean, SD	38.1 (6.4)	39.5 (6.9)	<0.001	39.40 (5.43)	39.48 (6.88)	0.592	39.22 (5.45)	39.48 (6.87)	0.130
Aaverage patient age during the hospitalization (years), mean, SD	71.2 (3.5)	71.3 (3.9)	0.484	71.15 (3.53)	71.29 (3.89)	0.143	71.15 (3.57)	71.25 (3.88)	0.317
meeting criteria for high-acuity caren (%), mean, SD	21.3 (9.9)	19.4 (9.9)	<0.001	19.07 (9.92)	19.39 (9.90)	0.231	18.92 (9.79)	19.40 (9.92)	0.075

[a] In the propensity score estimation, adjustments were made for age, age squared, Charlson Comorbidity Index (CCI), smoking index, body mass index (BMI), place of residence before admission, ICU stay, unconsciousness at admission, ambulance use, weekend admission, place after discharge (for the readmission model only), main diagnosis category, the Japanese Severity and Medical/Nursing Care Needs Score (Items A, B, and C) at admission, percentage of severe inpatients in the ward, median age of inpatients in the ward, number of inpatients in the ward, and hospital fixed effects (dummy variables).

[b] Chi-square test or t-test

[c] The Severity of a Patient's Condition and Extent of a Patient's Need for Medical/Nursing Care for General Wards comprises 21 items. Item A (7 items) assesses the need for specialized nursing care, including monitoring and treatment; Item B (7 items) evaluates patients' functional status, such as activities of daily living (ADLs), that influence health service use; and Item C (7 items) measures the need for medical management, including treatments related to surgery and emergency care.

© 2026 Morioka N et al. *JAMA Network Open*.

eTable 11. Sensitivity Analysis for In-Hospital Death, Readmission, and Length of Stay Using Propensity Score Matching With Planned Repeated Readmission Cases

	Before PS matching					After PS[a] matching				
	Adequately staffed group (>=annual median)		Understaffed group (below annual median)		P-value[b]	Adequately staffed group (>=annual median)		Understaffed group (below annual median)		P-value[b]
	Total Number	n (%)/ mean (SD)	Total Number	n (%)/ mean (SD)		Total Number	n (%)/ mean (SD)	Total Number	n (%)/ mean (SD)	
In hospital death										
Model 1: 24-hour period	48,579	1,161 (2.4)	37,485	1,173 (3.1)	<0.001	34,732	876 (2.7%)	34,732	964 (3.0%)	0.04
Model 2: Day shift	49,404	1,165 (2.4)	36,660	1,169 (3,2)	<0.001	27,335	936 (2.7%)	27,335	1,037 (3.0%)	0.02
Model 3: Evening and night shift	45,350	1,214 (2.7)	40,714	1,120 (2.8)	0.51	31,306	806 (2.9%)	31,306	767 (2.8%)	0.32
7-day readmission										
Model 1: 24-hour period	47,418	1,003 (2.1)	36,312	827 (2.3)	0.11	33,615	663 (2.1%)	33,615	717 (2.3%)	0.14
Model 2: Day shift	49,404	1,049 (2.1)	36,660	869 (2.4)	0.02	26,588	714 (2.1%)	26,588	779 (2.3%)	0.09
Model 3: Evening and night shift	45,350	973 (2.2)	40,714	945 (2.3)	0.08	29,631	579 (2.2%)	29,631	605 (2.3%)	0.45
30-day readmission	47,418	5,023 (10.6)	36,312	3,861 (10.6)	0.85	33,615	3,032 (10.2%)	33,615	3,191 (10.8%)	0.03
Model 1: 24-hour period	48,239	5,126 (10.6)	35,491	3,758 (10.6)	0.86	26,588	3,446 (10.3%)	26,588	3,593 (10.7%)	0.06
Model 2: Day shift	44,136	4,707 (10.7)	39,594	4,177 (10.6)	0.59	26,588	2,794 (10.5%)	26,588	2,846 (10.7%)	0.46
Model 3: Evening and night shift										
Length of stay										
Model 1: 24-hour period	48,579	12.42 (14.9)	37,485	14.85 (16.8)	<0.001	34,732	13.50 (15.99)	34,732	14.40 (16.08)	<0.001
Model 2: Day shift	49,404	12.41 (14.8)	36,660	14.91 (16.9)	<0.001	40,714	13.53 (15.85)	40,714	14.49 (16.21)	<0.001
Model 3: Evening and night shift	45,350	13.05 (15.7)	40,714	13.95 (15.9)	<0.001	40,714	13.41 (16.08)	40,714	13.95 (15.92)	<0.001

PS: Propensity score; SD: Standard deviation.

[a] In propensity score matching, adjusted for age, squared age, CCI, smoking index, BMI, prehospital residence, diagnosis, ICU stay, surgery, unconsciousness at admission, ambulance use, weekend admission, Severity of a Patient's Condition and Extent of a Patient's Need at admission, the percentage of severe inpatient on a ward, the median age of inpatient on a ward, the number of inpatients on a ward, dummy of hospital.

[b] Chi-square test or t-test

© 2026 Morioka N et al. *JAMA Network Open*.

eTable 12. Sensitivity Analysis for In-Hospital Death, Readmission, and Length of Stay Using Multilevel Models With Planned Repeated Readmission Cases

Understaffed relative to typical[a]	OR (95% CI)[b]	P-value
In hospital death (n=80,894)		
Model 1: 24-hour period	1.21 (1.11, 1.33)	<0.001
Model 2: Day shift	1.21 (1.11, 1.33)	<0.001
Model 3: Evening and night shift	1.02 (0.92, 1.12)	0.74
7-day readmission (n=83,568)		
Model 1: 24-hour period	1.06 (0.96, 1.17)	0.26
Model 2: Day shift	1.09 (0.99, 1.18)	0.07
Model 3: Evening and night shift	1.07 (0.96, 1.18)	0.22
30-day readmission (n=83,728)		
Model 1: 24-hour period	1.04 (0.99, 1.09)	0.08
Model 2: Day shift	1.04 (0.99, 1.09)	0.14
Model 3: Evening and night shift	1.00 (0.95, 1.05)	0.99
	Coef. (95% CI)	P-value
Length of stay (n=86,064)		
Model 1: 24-hour period	1.23 (1.03, 1.44)	<0.001
Model 2: Day shift	1.21 (1.02, 1.41)	<0.001
Model 3: Evening and night shift	-0.16 (-0.39, 0.07)	0.17

[a] The cutoff points for defining understaffing: the annual ward-level median relative to the median NHPPD for each model.

[b] Adjusted for age, squared age, CCI, smoking index, BMI, place of residence before admission, ICU stay, unconsciousness at admission, ambulance use, weekend admission, place after discharge (readmission model only), main diagnosis category, the Japanese Severity and Medical/Nursing Care Needs Score (Items A, B, and C) at admission, the percentage of severe inpatients in a ward, the median age of inpatients in a ward, the number of inpatients in a ward, and ward and hospital fixed effects (dummy variables).

© 2026 Morioka N et al. *JAMA Network Open*.

eTable 13. Sensitivity Analysis for Length of Stay Using Propensity Score Matching With and Without In-Hospital Deaths

Understaffed relative to typical[a]	With in-hospital death cases					Without in-hospital death cases				
	Adequately staffed group		Understaffed group		P-value[b]	Adequately staffed group		Understaffed group		P-value[b]
	Total Number	mean (SD)	Total Number	mean (SD)		Total Number	mean (SD)	Total Number	mean (SD)	
**Model 1: 24-hour period**	28,846	13.8 (16.5)	28,846	14.6 (16.3)	<0.001	27,940	13.2 (15.4)	27,940	14.1 (15.7)	<0.001
**Model 2: Day shift**	31,030	13.7 (16.2)	31,030	14.7 (16.4)	<0.001	30,028	13.2 (15.2)	30,028	14.2 (15.6)	<0.001
**Model 3: Evening/night shift**	36,272	13.6 (16.4)	36,272	14.1 (16.2)	<0.001	35,209	13.1 (15.4)	35,209	13.7 (15.5)	<0.001

[a] The cutoff points for defining understaffing: the annual ward-level median relative to the median NHPPD for each model.

[b] Chi-squared test or t-test was used, as appropriate.

In the propensity score estimation, adjustments were made for age, age squared, Charlson Comorbidity Index (CCI), smoking index, body mass index (BMI), place of residence before admission, ICU stay, unconsciousness at admission, ambulance use, weekend admission, place after discharge (for the readmission model only), main diagnosis category, the Japanese Severity and Medical/Nursing Care Needs Score (Items A, B, and C) at admission, percentage of severe inpatients in the ward, median age of inpatients in the ward, number of inpatients in the ward, and hospital fixed effects (dummy variables).

© 2026 Morioka N et al. *JAMA Network Open*.

eTable 14. Sensitivity Analysis for Length of Stay Using Multilevel Models With and Without In-Hospital Deaths

Understaffed relative to typical[a]	With in-hospital death cases[b]				Without in-hospital death cases[b]			
	Coef.	95% CI		P-value	Coef.	95% CI		P-value
**Model 1: 24-hour period**	1.22	1.00	1.43	<0.001	1.21	1.00	1.41	<0.001
**Model 2: Day shift**	1.22	1.01	1.44	<0.001	1.21	1.01	1.42	<0.001
**Model 3: Evening/night shift**	-0.16	-0.40	0.09	0.212	-0.05	-0.28	0.18	0.677

[a] The cutoff points for defining understaffing: the annual ward-level median relative to the median NHPPD for each model.

[b] Adjusted for age, squared age, CCI, smoking index, BMI, place of residence before admission, ICU stay, unconsciousness at admission, ambulance use, weekend admission, place after discharge (readmission model only), main diagnosis category, the Japanese Severity and Medical/Nursing Care Needs Score (Items A, B, and C) at admission, the percentage of severe inpatients in a ward, the median age of inpatients in a ward, the number of inpatients in a ward, and hospital fixed effects (dummy variables).

© 2026 Morioka N et al. *JAMA Network Open*.

eTable 15. Sensitivity Analysis for In-Hospital Death, Readmission, and Length of Stay Using Propensity Score Matching With or Without 15% Variation Around the Annual Median Cutoff

Cut-off point for defining understaffing[a]	After PS matching[b] for in-hospital death				P-value[c]
	Adequately staffed group		Understaffed group		
	Total Number	n (%)	Total Number	n (%)	
**Model 1: 24-hour period**					
Median+15%	10,379	209 (2.0%)	10,379	267 (2.6%)	0.007
Median+10%	16,925	350 (2.1%)	16,925	417 (2.5%)	0.014
Median+5%	25,770	625 (2.4%)	25,770	718 (2.8%)	0.010
Median	28,846	807 (2.8%)	28,846	905 (3.1%)	0.016
Median-5%	17,654	506 (2.9%)	17,654	552 (3.1%)	0.151
Median-10%	7,535	224 (3.0%)	7,535	228 (3.0%)	0.849
Median-15%	2,808	67 (2.4%)	2,808	96 (3.4%)	0.021
**Model 2: Day shift**					
Median+15%	15,124	300 (2.0%)	15,124	403 (2.7%)	<0.001
Median+10%	21,877	469 (2.1%)	21,877	576 (2.6%)	<0.001
Median+5%	29,549	701 (2.4%)	29,549	824 (2.8%)	0.001
Median	31,030	884 (2.8%)	31,030	983 (3.2%)	0.020
Median-5%	20,343	573 (2.8%)	20,343	671 (3.3%)	0.005
Median-10%	11,106	309 (2.8%)	11,106	364 (3.3%)	0.031
Median-15%	5,740	135 (2.4%)	5,740	208 (3.6%)	<0.001
**Model 3: Evening/night shift**					
Median+15%	7,079	172 (2.4%)	7,079	171 (2.4%)	0.956
Median+10%	12,352	288 (2.3%)	12,352	295 (2.4%)	0.769
Median+5%	19,782	497 (2.5%)	19,782	536 (2.7%)	0.219
Median	24,462	722 (3.0%)	24,462	750 (3.1%)	0.459
Median-5%	14,486	414 (2.9%)	14,486	390 (2.7%)	0.391
Median-10%	4,235	89 (2.1%)	4,235	95 (2.2%)	0.655
Median-15%	76,551	2,191 (2.9%)	76,551	13 (1.8%)	0.076

Cut-off point for defining understaffing[a]	After PS matching[b] for 7-day readmission				P-value[c]
	Adequately staffed group		Understaffed group		
	Total Number	n (%)	Total Number	n (%)	
**Model 1: 24-hour period**					
Median+15%	10,170	195 (1.9%)	10,170	225 (2.2%)	0.139
Median+10%	16,526	312 (1.9%)	16,526	378 (2.3%)	0.011
Median+5%	25,061	503 (2.0%)	25,061	563 (2.2%)	0.063
Median	27,907	616 (2.2%)	27,907	636 (2.3%)	0.568
Median-5%	17,087	404 (2.4%)	17,087	379 (2.2%)	0.366
Median-10%	7,305	175 (2.4%)	7,305	159 (2.2%)	0.376
Median-15%	2,712	55 (2.0%)	2,712	59 (2.2%)	0.705
**Model 2: Day shift**					
Median+15%	14,823	281 (1.9%)	14,823	327 (2.2%)	0.059
Median+10%	21,365	401 (1.9%)	21,365	494 (2.3%)	0.002

Median+5%	28,794	561 (1.9%)	28,794	657 (2.3%)	0.005
Median	29,999	628 (2.1%)	29,999	701 (2.3%)	0.043
Median-5%	19,672	452 (2.3%)	19,672	423 (2.2%)	0.321
Median-10%	10,739	227 (2.1%)	10,739	236 (2.2%)	0.672
Median-15%	5,532	112 (2.0%)	5,532	104 (1.9%)	0.583
**Model 3: Evening/night shift**					
Median+15%	6,901	135 (2.0%)	6,901	145 (2.1%)	0.546
Median+10%	12,018	251 (2.1%)	12,018	247 (2.1%)	0.856
Median+5%	19,247	392 (2.0%)	19,247	390 (2.0%)	0.942
Median	23,737	538 (2.3%)	23,737	529 (2.2%)	0.780
Median-5%	14,083	298 (2.1%)	14,083	300 (2.1%)	0.934
Median-10%	3,987	73 (1.8%)	3,987	83 (2.1%)	0.419
Median-15%	6,901	135 (2.0%)	6,901	145 (2.1%)	0.546

Cut-off point for defining understaffing[a]	After PS matching[b] for 30-day readmission				P-value[c]
	Adequately staffed group		Understaffed group		
	Total Number	n (%)	Total Number	n (%)	
**Model 1: 24-hour period**					
Median+15%	10,170	1,115 (11.0%)	10,170	1,169 (11.5%)	0.230
Median+10%	16,526	1,811 (11.0%)	16,526	1,921 (11.6%)	0.056
Median+5%	25,061	2,693 (10.7%)	25,061	2,897 (11.6%)	0.004
Median	27,907	2,942 (10.5%)	27,907	3,126 (11.2%)	0.012
Median-5%	17,087	1,790 (10.5%)	17,087	1,859 (10.9%)	0.227
Median-10%	7,305	765 (10.5%)	7,305	788 (10.8%)	0.537
Median-15%	2,712	276 (10.2%)	2,712	281 (10.4%)	0.823
**Model 2: Day shift**					
Median+15%	14,823	1,623 (10.9%)	14,823	1,706 (11.5%)	0.127
Median+10%	21,365	2,320 (10.9%)	21,365	2,416 (11.3%)	0.139
Median+5%	28,794	3,086 (10.7%)	28,794	3,254 (11.3%)	0.025
Median	29,999	3,169 (10.6%)	29,999	3,306 (11.0%)	0.071
Median-5%	19,672	2,030 (10.3%)	19,672	2,109 (10.7%)	0.194
Median-10%	10,739	1,100 (10.2%)	10,739	1,167 (10.9%)	0.137
Median-15%	5,532	554 (10.0%)	5,532	581 (10.5%)	0.398
**Model 3: Evening/night shift**					
Median+15%	6,901	795 (11.5%)	6,901	779 (11.3%)	0.668
Median+10%	12,018	1,324 (11.0%)	12,018	1,364 (11.3%)	0.413
Median+5%	19,247	2,110 (11.0%)	19,247	2,122 (11.0%)	0.845
Median	23,737	2,601 (11.0%)	23,737	2,637 (11.1%)	0.598
Median-5%	14,083	1,520 (10.8%)	14,083	1,541 (10.9%)	0.688
Median-10%	3,987	409 (10.3%)	3,987	457 (11.5%)	0.084
Median-15%	74,360	8,095 (10.9%)	74,360	87 (12.0%)	0.318

© 2026 Morioka N et al. *JAMA Network Open*.

Cut-off point for defining understaffing[a]	After PS matching[b] for LOS				P-value[c]
	Adequately staffed group		Understaffed group		
	Total Number	mean (SD)	Total Number	mean (SD)	
**Model 1: 24-hour period**					
Median+15%	10,379	8.6 (11.4)	10,379	11.6 (13.0)	<0.001
Median+10%	16,925	9.7 (11.9)	16,925	12.2 (13.6)	<0.001
Median+5%	25,770	11.3 (13.7)	25,770	13.1 (14.6)	<0.001
Median	28,846	13.8 (16.5)	28,846	14.6 (16.3)	<0.001
Median-5%	17,654	14.7 (17.7)	17,654	12.3 (13.6)	<0.001
Median-10%	7,535	14.0 (16.9)	7,535	10.3 (9.9)	<0.001
Median-15%	2,808	13.0 (15.0)	2,808	8.2 (7.7)	<0.001
**Model 2: Day shift**					
Median+15%	15,124	9.4 (11.6)	15,124	12.2 (14.2)	<0.001
Median+10%	21,877	10.4 (12.6)	21,877	12.7 (14.6)	<0.001
Median+5%	29,549	11.9 (14.3)	29,549	13.5 (15.2)	<0.001
Median	31,030	13.7 (16.2)	31,030	14.7 (16.4)	<0.001
Median-5%	20,343	14.4 (17.5)	20,343	13.5 (14.6)	<0.001
Median-10%	11,106	14.5 (17.9)	11,106	11.3 (11.2)	<0.001
Median-15%	5,740	13.7 (16.1)	5,740	9.4 (9.1)	<0.001
**Model 3: Evening/night shift**					
Median+15%	7,079	9.4 (12.3)	7,079	11.2 (12.8)	<0.001
Median+10%	12,352	10.1 (12.6)	12,352	11.6 (13.1)	<0.001
Median+5%	19,782	11.7 (13.9)	19,782	12.8 (14.7)	<0.001
Median	36,272	13.6 (16.4)	36,272	14.1 (16.2)	<0.001
Median-5%	14,486	14.3 (17.0)	14,486	11.4 (11.3)	<0.001
Median-10%	4,235	12.4 (15.3)	4,235	8.9 (8.4)	<0.001
Median-15%	76,551	13.7 (16.1)	76,551	7.1 (6.8)	<0.001

[a] There are seven cutoff points for defining understaffing: the annual ward-level median and alternative points (+15%, +10%, +5%, -5%, -10%, and -15% relative to the median NHPPD) for each model.

[b] In the propensity score estimation, adjustments were made for age, age squared, Charlson Comorbidity Index (CCI), smoking index, body mass index (BMI), place of residence before admission, ICU stay, unconsciousness at admission, ambulance use, weekend admission, place after discharge (for the readmission model only), main diagnosis category, the Japanese Severity and Medical/Nursing Care Needs Score (Items A, B, and C) at admission, percentage of severe inpatients in the ward, median age of inpatients in the ward, number of inpatients in the ward, and hospital fixed effects (dummy variables).

[c] Chi-square test or t-test

© 2026 Morioka N et al. *JAMA Network Open*.

eTable 16. Sensitivity Analysis for In-Hospital Death, Readmission, and Length of Stay Using Multilevel Models With and Without 15% Variation Around the Annual Median Cutoff

Cut-off point for defining understaffing[a]	In hospital death[b] n=72,119				7-day readmission n=75,238				30-day readmission n=75,238				LOS n=74,921			
	Adjusted OR[c]	95% CI		P-value	Adjusted OR[c]	95% CI		P-value	Adjusted OR[c]	95% CI		P-value	Coef.[c]	95% CI		P-value
**Model 1: 24-hour period**																
Median+15%	1.26	1.07	1.48	0.006	1.10	0.94	1.29	0.236	1.07	0.99	1.15	0.083	3.32	2.99	3.65	<0.001
Median+10%	1.30	1.15	1.48	<0.001	1.15	1.01	1.31	0.031	1.04	0.98	1.10	0.219	3.26	2.99	3.53	<0.001
Median+5%	1.24	1.12	1.38	<0.001	1.11	0.99	1.23	0.065	1.05	1.00	1.11	0.063	2.55	2.32	2.78	<0.001
Median	1.22	1.11	1.34	<0.001	1.07	0.96	1.18	0.222	1.05	1.00	1.10	0.053	1.22	1.00	1.43	<0.001
Median-5%	1.17	1.05	1.30	0.005	1.02	0.91	1.15	0.702	1.03	0.98	1.10	0.266	-1.56	-1.81	-1.31	<0.001
Median-10%	1.23	1.06	1.44	0.008	1.02	0.86	1.20	0.853	1.01	0.93	1.10	0.795	-3.72	-4.07	-3.37	<0.001
Median-15%	1.56	1.24	1.97	<0.001	1.02	0.78	1.34	0.857	1.00	0.88	1.14	0.970	-5.09	-5.64	-4.54	<0.001
**Model 2: Day shift**																
Median+15%	1.36	1.19	1.56	<0.001	1.15	1.00	1.32	0.044	1.06	0.99	1.12	0.087	3.20	2.93	3.47	<0.001
Median+10%	1.32	1.18	1.48	<0.001	1.20	1.07	1.35	0.002	1.06	1.00	1.12	0.048	2.89	2.65	3.12	<0.001
Median+5%	1.26	1.14	1.39	<0.001	1.14	1.03	1.26	0.015	1.05	1.00	1.11	0.040	2.14	1.93	2.36	<0.001
Median	1.20	1.10	1.32	<0.001	1.12	1.01	1.24	0.029	1.04	0.99	1.10	0.084	1.22	1.01	1.44	<0.001
Median-5%	1.22	1.10	1.35	<0.001	0.98	0.88	1.10	0.768	1.02	0.97	1.08	0.442	-0.88	-1.11	-0.64	<0.001
Median-10%	1.29	1.14	1.46	<0.001	1.02	0.89	1.18	0.780	1.03	0.96	1.11	0.361	-2.87	-3.16	-2.58	<0.001
Median-15%	1.53	1.31	1.80	<0.001	0.86	0.71	1.06	0.160	1.01	0.93	1.11	0.757	-4.42	-4.81	-4.03	<0.001
**Model 3: Evening/night shift**																
Median+15%	0.98	0.81	1.18	0.837	1.08	0.89	1.31	0.438	0.98	0.9	1.07	0.664	2.32	1.91	2.73	<0.001
Median+10%	1.05	0.90	1.22	0.527	0.99	0.86	1.15	0.938	1.02	0.95	1.09	0.631	2.59	2.26	2.92	<0.001
Median+5%	1.12	1.00	1.26	0.050	1.08	0.96	1.22	0.225	0.98	0.92	1.04	0.425	2.25	1.98	2.52	<0.001
Median	1.04	0.94	1.15	0.433	1.04	0.94	1.16	0.467	1.00	0.94	1.05	0.909	-0.16	-0.40	0.09	0.212
Median-5%	0.96	0.85	1.09	0.527	0.96	0.84	1.09	0.543	0.98	0.92	1.05	0.624	-3.72	-4.00	-3.43	<0.001
Median-10%	0.97	0.77	1.23	0.824	0.97	0.78	1.22	0.824	1.01	0.91	1.12	0.854	-4.58	-5.06	-4.10	<0.001
Median-15%	0.74	0.41	1.36	0.338	1.09	0.66	1.79	0.735	1.05	0.83	1.33	0.668	-5.14	-6.21	-4.07	<0.001

[a] There are seven cutoff points for defining understaffing: the annual ward-level median and alternative points (+15%, +10%, +5%, -5%, -10%, and -15% relative to the median NHPPD) for each model.

[b] In the propensity score estimation, adjustments were made for age, age squared, Charlson Comorbidity Index (CCI), smoking index, body mass index (BMI), place of residence before admission, ICU stay, unconsciousness at admission, ambulance use, weekend admission, place after discharge (for the readmission model only), main diagnosis category, the Japanese Severity and Medical/Nursing Care Needs Score (Items A, B, and C) at admission, percentage of severe inpatients in the ward, median age of inpatients in the ward, number of inpatients in the ward, and hospital fixed effects (dummy variables).

© 2026 Morioka N et al. *JAMA Network Open*.

eFigure 1. Flowchart of Sample Selection

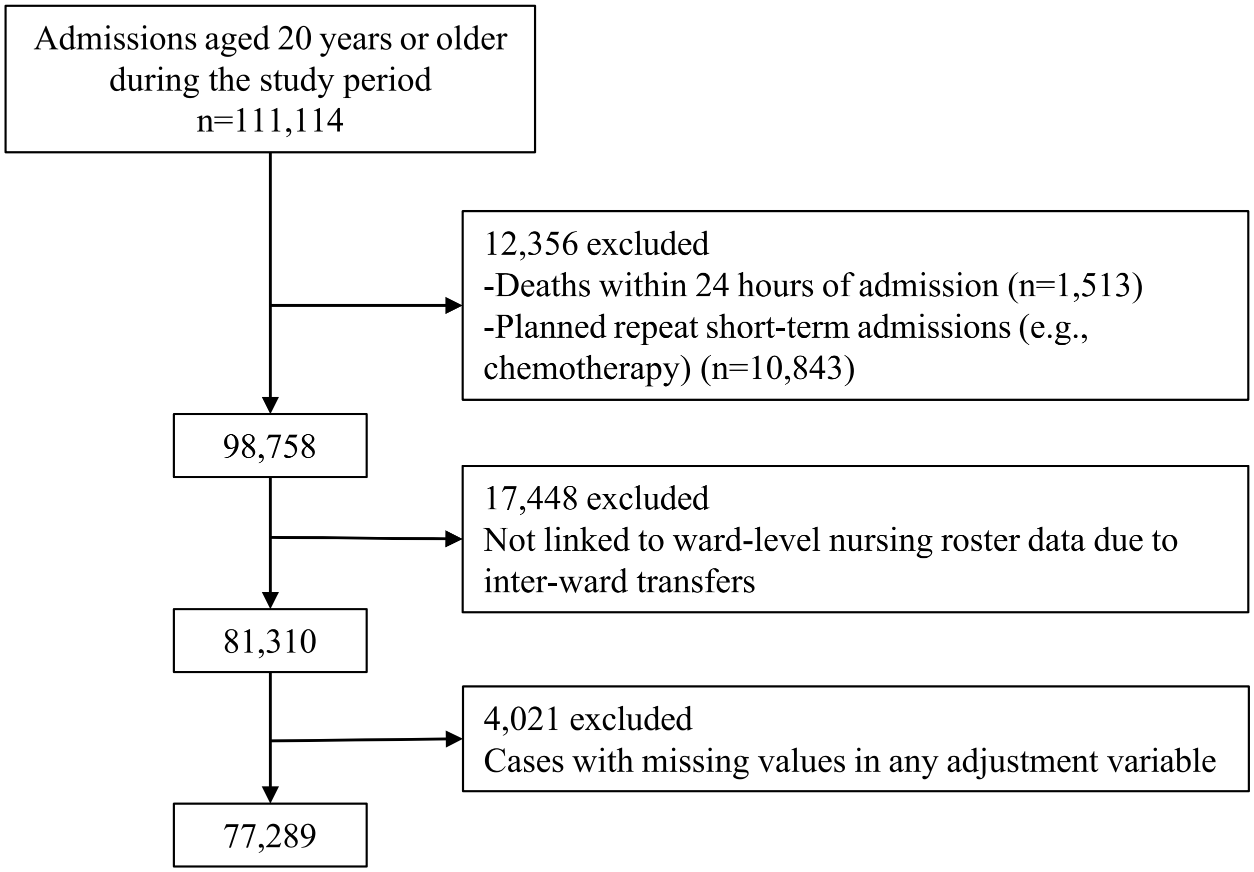

© 2026 Morioka N et al. *JAMA Network Open*.

eFigure 2. Nurse Staffing Levels for the 24-Hour Period, Day Shift, and Evening and Night Shift

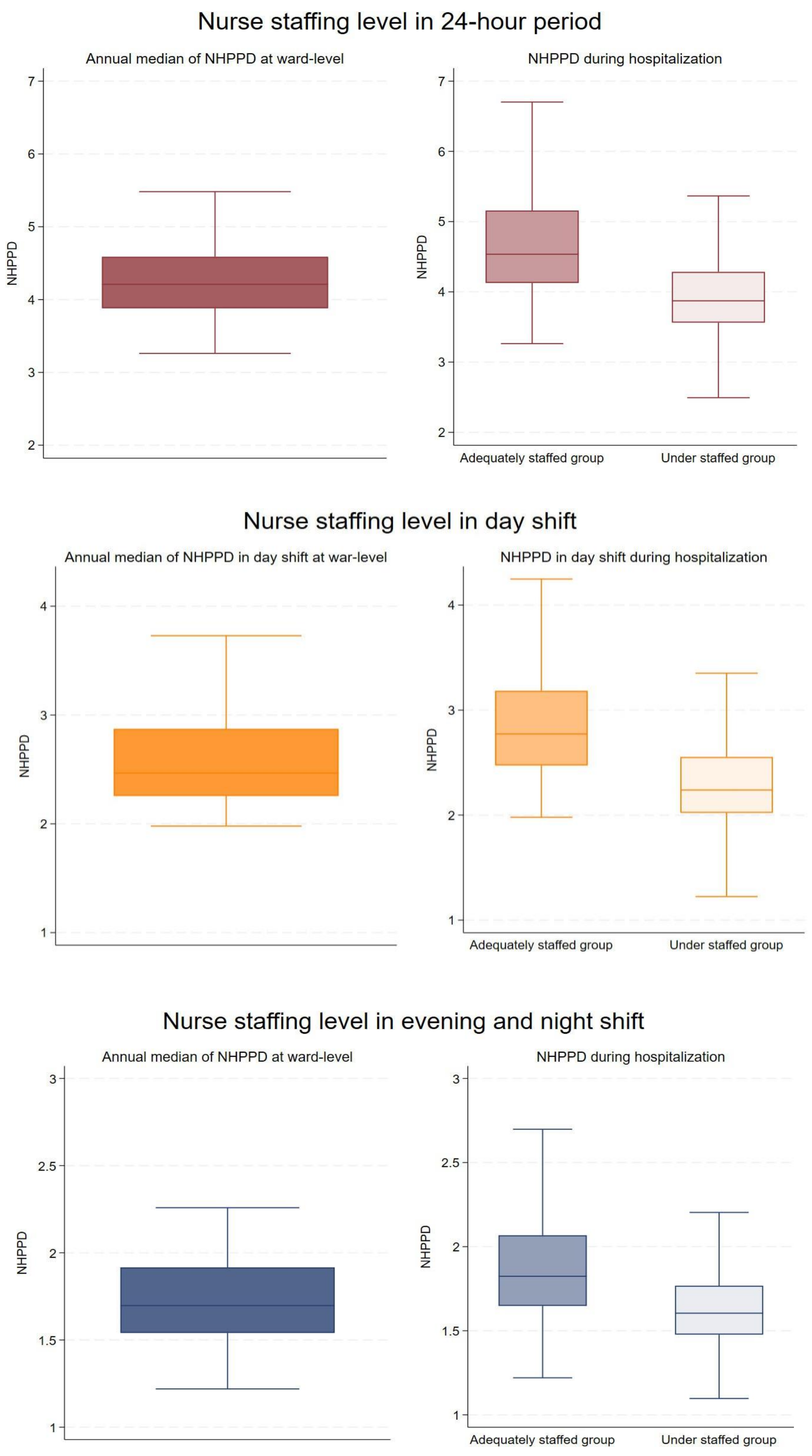

© 2026 Morioka N et al. *JAMA Network Open*.
