# Supplementary material for: Hospital Nurse Understaffing and Patient Mortality, Readmission, and Length of Stay
Source: JAMA Netw Open. 2026 Feb 25;9(2):e2558235. doi: 10.1001/jamanetworkopen.2025.58235 (PMC12936879; doi:10.1001/jamanetworkopen.2025.58235)
Supplement: Supplement 2. — Data Sharing Statement [file jamanetwopen-e2558235-s002.pdf]

Data Sharing Statement

Morioka. Hospital Nurse Understaffing and Patient Mortality, Readmission, and Length of Stay. *JAMA Netw Open*. Published February 25, 2026. doi:10.1001/jamanetworkopen.2025.58235

Data
Data available: No
